# Spermine and spermidine reversed age-related cardiac deterioration in rats

**DOI:** 10.18632/oncotarget.18334

**Published:** 2017-05-31

**Authors:** Hao Zhang, Junying Wang, Lingxu Li, Nannan Chai, Yuhan Chen, Feixiang Wu, Weihua Zhang, Lina Wang, Sa Shi, Li Zhang, Shuling Bian, Changqing Xu, Ye Tian, Yajun Zhao

**Affiliations:** ^1^ Department of Pathophysiology, The Key Laboratory of Cardiovascular Pathophysiology, Harbin Medical University, Harbin, China; ^2^ College of Nursing, Medical School of Chifeng University, Chifeng, China; ^3^ Experiment Center of Function, Harbin Medical University, Harbin, China; ^4^ Key Laboratory of Cardiovascular Medicine Research, Harbin Medical University, Ministry of Education, Harbin, China

**Keywords:** polyamines, proteomics, metabolomics, aging, cardiovascular disease, Gerotarget

## Abstract

Aging is the most important risk factor for cardiovascular disease (CVD). Slowing or reversing the physiological impact of heart aging may reduce morbidity and mortality associated with age-related CVD. The polyamines, spermine (SP) and spermidine (SPD) are essential for cell growth, differentiation and apoptosis, and levels of both decline with age. To explore the effects of these polyamines on heart aging, we administered SP or SPD intraperitoneally to 22- to 24-month-old rats for 6 weeks. Both treatments reversed and inhibited age-related myocardial morphology alterations, myocardial fibrosis, and cell apoptosis. Using combined proteomics and metabolomics analyses, we identified proteins and metabolites up- or downregulated by SP and SPD in aging rat hearts. SP upregulated 51 proteins and 28 metabolites while downregulating 80 proteins and 29 metabolites. SPD upregulated 44 proteins and 24 metabolites and downregulated 84 proteins and 176 metabolites. These molecules were mainly associated with immune responses, blood coagulation, lipid metabolism, and glutathione metabolism pathways. Our study provides novel molecular information on the cardioprotective effects of polyamines in the aging heart, and supports the notion that SP and SPD are potential clinical therapeutics targeting heart disease.

## INTRODUCTION

Age-related diseases are major contributors to high morbidity and mortality rates in aging populations. Cardiovascular diseases (CVDs), such as coronary atherosclerosis, heart failure, and dilated cardiomyopathy, are age-associated diseases and leading causes of death worldwide. Identifying efficacious age-related CVD treatments is essential to extending elderly patient lifespans.

Polyamines (PAs) are small, linear or occasionally branched polycations derived from amino acids, and are found in almost all eukaryotic cells. They are essential for cell proliferation, differentiation, and apoptosis [1, 2], and function as anti-inflammatories, anti-oxidants, and free radical scavengers [3-5]. During the aging process, polyamines, especially spermidine (SPD), are depleted in the spleen, ovary, liver, stomach, lung, kidney, muscle, and thymus [6, 7]. Stegehake, *et al.* reported that cellular polyamine levels decreased in aging post-reproductive *Caenorhabditis elegans* [6]. Enot, *et al.* found that polyamines are depleted by palmitate, but enhanced by oleate in mouse liver, heart, and skeletal muscle [7]. Endogenous polyamines may extend cellular longevity by inducing autophagy [8-11]. Accumulating evidence indicates that polyamine levels are higher in the immature heart and decrease with age in male rats [12]. Spermine (SP) attenuates cardiac endoplasmic reticulum stress during acute myocardial infarction in male rat hearts by inhibiting reactive oxygen species (ROS) and downregulating PERK-eIF2α signaling [13-15]. Our previous study suggested that exogenous polyamine protects against reperfusion injury by inhibiting mitochondrial permeability transition pore (mPTP) opening in isolated rat hearts [14]. Additionally, exercise training can increase the polyamine pool in aged rat hearts, restoring ischemic preconditioning protection [15]. Recent work showed that SPD reduces lipid accumulation and necrotic core formation in atherosclerotic plaques by inducing autophagy in apolipoprotein E (ApoE) -/- mice [16]. SPD feeding enhanced cardiac mitophagy and mitochondrial respiration, and improved diastolic function to delay cardiac aging in C57BL/6 mice. Oral SPD supplementation inversely correlated with CVD in humans [17]. However, to the best of our knowledge, no definitive information is available regarding the effect of exogenous polyamines on rat heart aging.

Proteins are the primary effector molecules of all living systems, and any adaptive responses to exogenous stresses will be reflected in altered protein activities and concentrations [18]. Proteomics allows for direct study of protein production and function in a cellular context [19]. Isobaric tags for relative and absolute quantitation (iTRAQ) in combination with liquid chromatography tandem MS (LC-MS/MS) analysis is a new high throughput, high repeatability, high sensitivity, and high accuracy proteomics tool for studying biological processes [20, 21]. Proteomics analyses of aged rat hearts revealed changes in cell signaling, immune response, and structural proteins, and in proteins mediating oxidative stress responses [22]. Such changes can directly indicate aberrant physiological status [23]. Thus, proteomics and metabolomics technologies, which provide global profile information, are powerful tools for investigating tissue responses to drug treatment.

This study employed iTRAQ proteomic and GC/MS-based metabolomic approaches to investigate alterations in aging rat hearts treated with SP and SPD. In analyzing the pathways involved in SP and SPD activity, we provide novel insight into the cardioprotective mechanisms of these two PAs in aging rats.

## RESULTS

### Myocardial histology and morphology changes in SP- or SPD-treated aged rats

**Figure 1:**
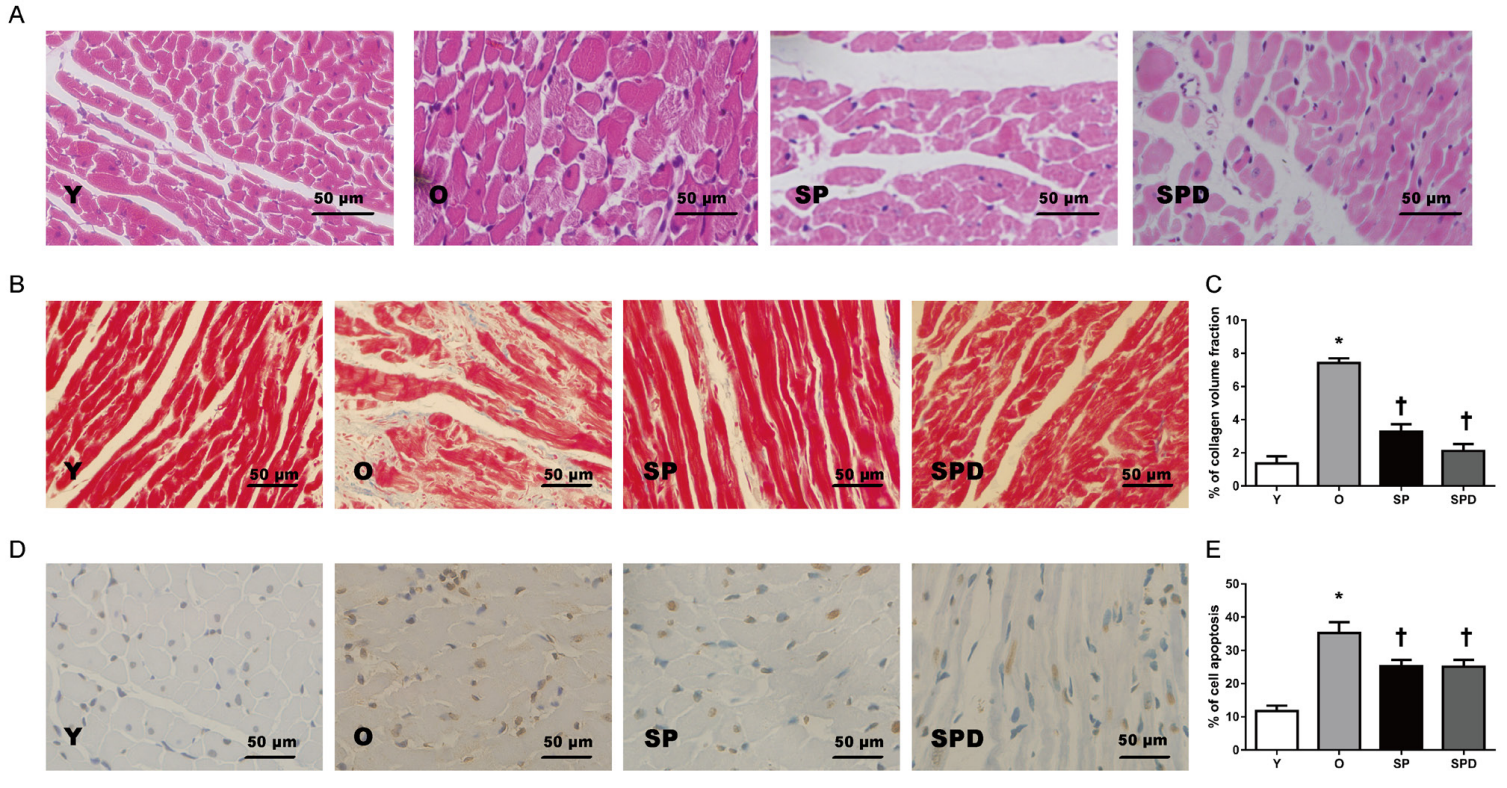
Myocardial histology and morphology in young (Y), old (O), spermine (SP)-, and spermidine (SPD)-treated rats Representative left ventricle midwall sections stained by H&E (40×) **A.** Masson’s trichrome staining and CVF represent interstitial fibrotic areas in left ventricle midwall sections in Y, O, and SP- or SPD-treated rats **B.** & **C.** *n* = 6 per group. Representative illustration of TUNEL staining in cardiomyocytes from different groups **D.** Nuclei with brown staining indicate TUNEL-positive cells (400×). Percentage of TUNEL-positive nuclei and total nuclei in different groups **E**. *n* = 6 per group, *P < 0.05 *vs.* Y group; †*P* < 0.05 *vs*. O group.

Age-related phenotype changes were observed in rat hearts, including myocardial morphology changes, myocardial fibrosis, and cell apoptosis. Compared to young control rats (group Y), aged rat hearts showed loosely arranged cardiac muscle fibers and increased interstitial distances as evaluated by H&E staining. However, hearts in aged rats treated with SP or SPD maintained myocardial histological structures as compared to untreated controls (group O) (Figure 1A). Increased myocardial collagen deposition and a disordered collagen fiber network around cardiomyocytes were observed in old rat hearts (Figure 1B), and collagen volume fraction (CVF) was higher than in young rats (*P* < 0.05). However, CVF was decreased to a greater extent in SP- (*P* < 0.05) or SPD-treated (*P* < 0.05) old rat hearts compared to untreated old rat hearts (Figure 1C). The ratio of TUNEL-positive cardiomyocytes to total cardiomyocytes was higher in old rat hearts than in young rat hearts (*P* < 0.05). In contrast, the positive cell ratio in SP- (*P* < 0.05) or SPD-treated (*P* < 0.05) rats was lower than in untreated old rats (Figure 1D-1E). These findings suggested that SP and SPD effectively reduced age-related cardiovascular changes in rats.

### Polyamine-regulated proteins in aged rat hearts

**Figure 2:**
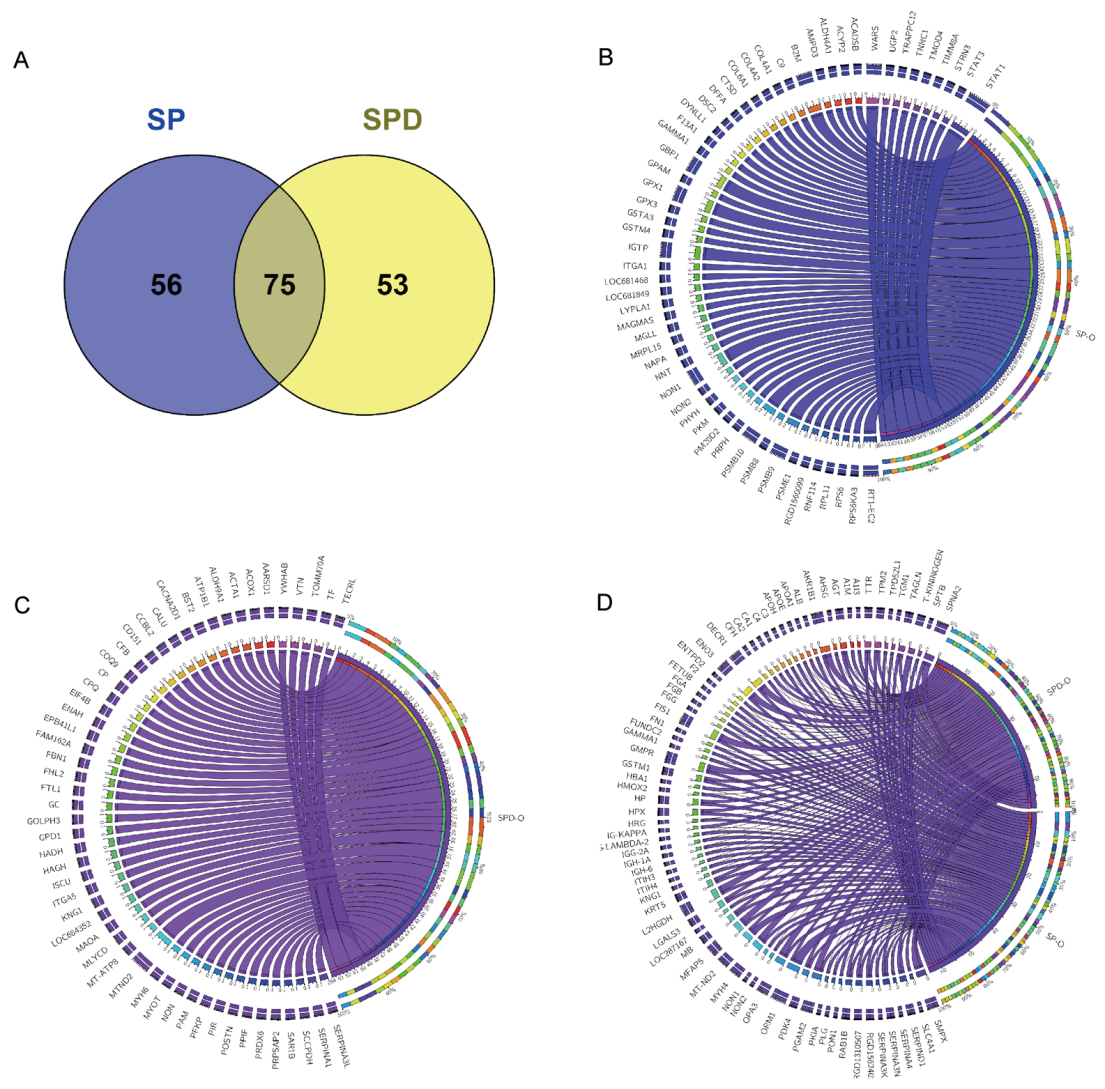
184 proteins differentially expressed in aged rat hearts following SP or SPD treatment Venn diagram of proteins differentially expressed between SP and SPD groups **A.** Circos plot for proteins differentially expressed between SP and SPD treatment groups. Groups and proteins are represented by circular segments with lengths proportional to the total fold change value. Fold change is represented by ribbons connecting groups and proteins. The three outer rings are stacked bar plots representing the relative contribution of a cell to group and protein totals. 56/131 proteins were differentially expressed in the SP group **B.**, 53/128 were differentially expressed in the SPD group **C.**, and 75were differentially expressed in both SP and SPD groups **D**.

To understand the effects of SP and SPD on protein expression in aged rat hearts, iTRAQ in combination with liquid chromatography-electrospray ionization tandem MS (LC-ESI-MS/MS) was applied to investigate differentially expressed proteins in treated and untreated rats. A total of 2586 protein groups were identified and quantified from 14401 unique peptides. Among these, 2565 protein groups were shared among the three complete iTRAQ datasets (SP, SPD, and O), which was proportionally consistent with reports on technical variance between iTRAQ datasets [22]. Of the 2565 proteins, 184 were differentially expressed in the SP and SPD groups compared with the old group (Supplementary Table 1) and were plotted against the old group using the circos plot (Figure 2). Of these, 131 proteins were differentially expressed in SP-treated rat hearts compared to untreated old rat hearts. Eighty proteins were downregulated, and 51 were upregulated (Figure 2B). Similarly, 128 of the 184 proteins were differentially expressed in SPD-treated rat hearts; 84 were downregulated and 44 were upregulated compared to untreated old rat hearts (Figure 2C). Seventy-five of 184 proteins were affected by both SP and SPD treatment (Figure 2D).

**Figure 3:**
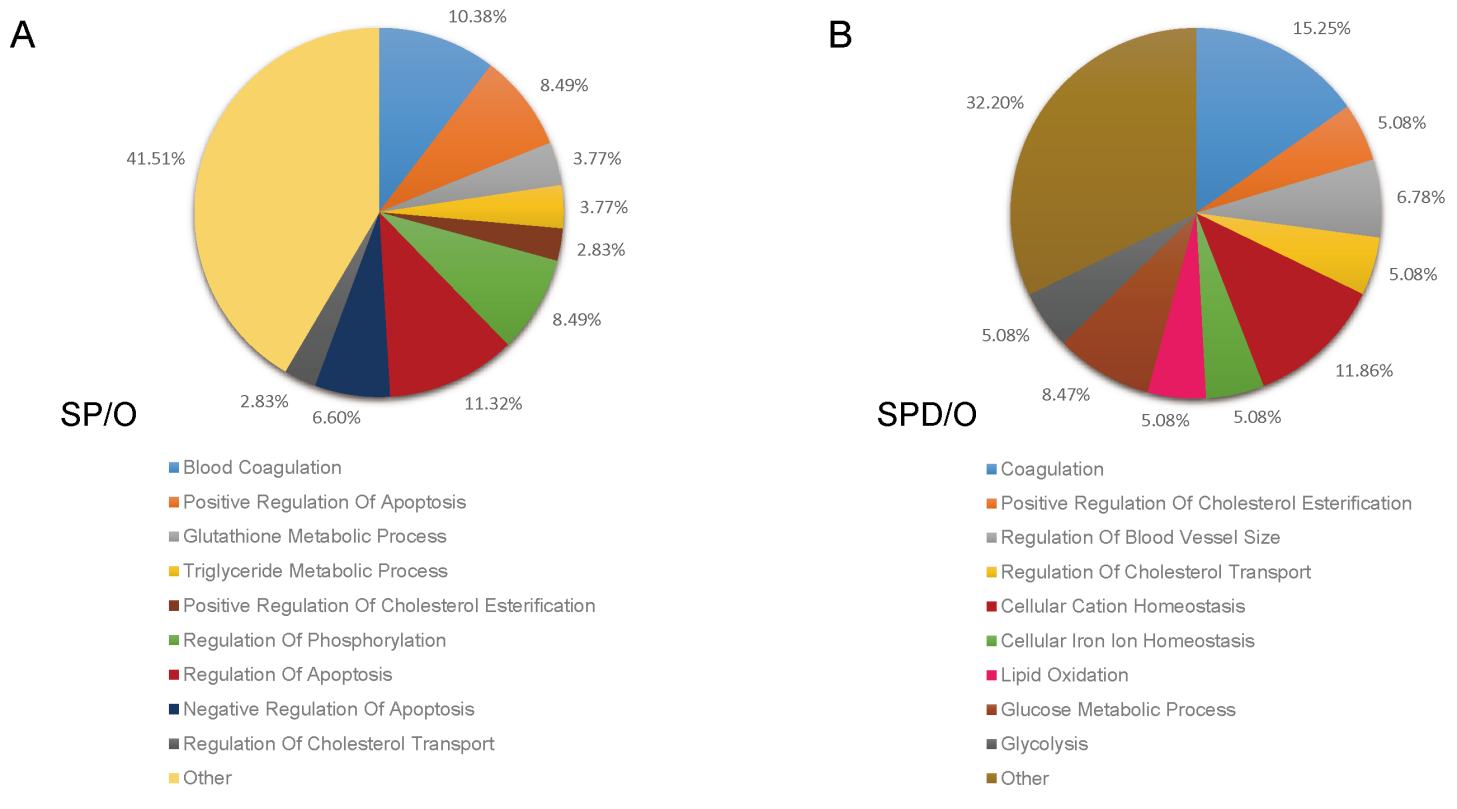
Functional classification of SP- and SPD-regulated proteins in aged rat hearts The DAVID bioinformatics platform clustered the 184 altered proteins by biological process. SP *vs.* O (**A**.) and SPD *vs*. O (**B.)**.

GO analysis was performed for proteins differentially expressed following polyamine treatment. Of the 131 differentially expressed proteins in SP-treated rat hearts, 112 clustered into categories with *P* < 0.05, including blood coagulation, positive regulation of apoptosis, triglyceride metabolic process, glutathione metabolic process, positive regulation of cholesterol esterification, and regulation of phosphorylation (Figure 3A, Table 1). In SPD-treated rats, 106 differentially expressed proteins clustered into the coagulation, positive regulation of cholesterol esterification, regulation of blood vessel size, lipid oxidation, glucose metabolic process, and glycolysis categories (Figure 3B, Table 2).

**Table 1:** GO analysis of 131 proteins from SP group

Category	Term	BP^a^	Count^b^	*P* Value^c^	Genes
GOTERM_BP_FAT	GO:0007596	blood coagulation	11	4.69E-11	FGG, C9, FGA, FGB, C3, F13A1, F2, APOH, SERPIND1, ENTPD2, PLG
GOTERM_BP_FAT	GO:0043065	positive regulation of apoptosis	9	0.003141	GPX1, C9, DYNLL1, APOE, AGT, ITGA1, RPS6, STAT1, PLG
GOTERM_BP_FAT	GO:0006749	glutathione metabolic process	4	0.001267	GSTM1, GPX1, GSTA3, GPX3
GOTERM_BP_FAT	GO:0006641	triglyceride metabolic process	4	0.006071	GPX1, APOE, APOH, GPAM
GOTERM_BP_FAT	GO:0010873	positive regulation of cholesterol esterification	3	8.06E-04	APOA1, APOE, AGT
GOTERM_BP_FAT	GO:0042325	regulation of phosphorylation	9	0.009622	KNG1, APOA1, DYNLL1, HPX, APOE, AGT, F2, ITGA1, PKIA
GOTERM_BP_FAT	GO:0042981	regulation of apoptosis	12	0.011547	GPX1, C9, DYNLL1, ALB, APOE, AGT, APOH, ITGA1, RPS6, STAT1, PLG, FN1
GOTERM_BP_FAT	GO:0043066	negative regulation of apoptosis	7	0.037712	GPX1, ALB, APOE, AGT, APOH, RPS6, FN1
GOTERM_BP_FAT	GO:0032374	regulation of cholesterol transport	3	0.006929	APOA1, APOE, PON1
GOTERM_BP_FAT	GO:0042246	tissue regeneration	4	0.004514	GPX1, FGA, ENO3, PLG
GOTERM_BP_FAT	GO:0009062	fatty acid catabolic process	3	0.029299	ACADSB, DECR1, PHYH
GOTERM_BP_FAT	GO:0031145	anaphase-promoting complex-dependent proteasomal ubiquitin-dependent protein catabolic process	4	0.007521	PSMB10, PSME1, PSMB8, PSMB9
GOTERM_BP_FAT	GO:0051605	protein maturation by peptide bond cleavage	4	0.016436	C9, C3, CFH, APOH
GOTERM_BP_FAT	GO:0035150	regulation of tube size	6	3.01E-05	KNG1, GPX1, ALB, APOE, AGT, ITGA1
GOTERM_BP_FAT	GO:0002821	positive regulation of adaptive immune response	3	0.033971	HPX, C3, B2M
GOTERM_BP_FAT	GO:0006956	complement activation	3	0.024909	C9, C3, CFH
GOTERM_BP_FAT	GO:0051258	protein polymerization	3	0.020819	FGG, FGA, FGB
GOTERM_BP_FAT	GO:0000278	mitotic cell cycle	6	0.026179	PSMB10, PSME1, TPD52L1, RPS6, PSMB8, PSMB9
GOTERM_BP_FAT	GO:0010740	positive regulation of protein kinase cascade	5	0.037932	KNG1, GPX1, HPX, AGT, AKR1B1
GOTERM_BP_FAT	GO:0010594	regulation of endothelial cell migration	3	0.006929	APOE, AGT, APOH

Note: Generated by DAVID 6.7 (https://david-d.ncifcrf.gov/).

^a^ Biology process.

^b^ The number of proteins derived from proteomics analysis.

^c^ Probability of identified differential protein list associates to the GO term.

**Table 2:** GO analysis of 128 proteins from SPD group

Category	Term	BP^a^	Count^b^	*P* Value^c^	Genes
GOTERM_BP_FAT	GO:0050817	coagulation	9	2.32E-08	FGG, FGA, FGB, C3, F2, APOH, SERPIND1, ENTPD2, PLG
GOTERM_BP_FAT	GO:0010873	positive regulation of cholesterol esterification	3	7.71E-04	APOA1, APOE, AGT
GOTERM_BP_FAT	GO:0050880	regulation of blood vessel size	4	0.00539	KNG1, ALB, APOE, AGT
GOTERM_BP_FAT	GO:0032374	regulation of cholesterol transport	3	0.006633	APOA1, APOE, PON1
GOTERM_BP_FAT	GO:0051605	protein maturation by peptide bond cleavage	4	0.015482	CFB, C3, CFH, APOH
GOTERM_BP_FAT	GO:0030003	cellular cation homeostasis	7	0.006992	KNG1, RGD1310507, TF, APOE, AGT, F2, CP
GOTERM_BP_FAT	GO:0006879	cellular iron ion homeostasis	3	0.022546	RGD1310507, TF, CP
GOTERM_BP_FAT	GO:0034440	lipid oxidation	3	0.035742	ACOX1, MLYCD, DECR1
GOTERM_BP_FAT	GO:0006006	glucose metabolic process	5	0.039378	GPD1, PDK4, PFKP, ENO3, PGAM2
GOTERM_BP_FAT	GO:0006096	glycolysis	3	0.042357	PFKP, ENO3, PGAM2
GOTERM_BP_FAT	GO:0006956	complement activation	3	0.023889	CFB, C3, CFH
GOTERM_BP_FAT	GO:0046165	alcohol biosynthetic process	3	0.040663	GPD1, AKR1B1, PGAM2
GOTERM_BP_FAT	GO:0051258	protein polymerization	3	0.019959	FGG, FGA, FGB
GOTERM_BP_FAT	GO:0051224	negative regulation of protein transport	3	0.02667	APOA1, YWHAB, PKIA
GOTERM_BP_FAT	GO:0010594	regulation of endothelial cell migration	3	0.006633	APOE, AGT, APOH

Note: Generated by DAVID 6.7 (https://david-d.ncifcrf.gov/).

^a^ Biology process.

^*b*^ The number of proteins derived from proteomics analysis.

^*c*^ Probability of identified differential protein list associates to the GO term.

**Figure 4:**
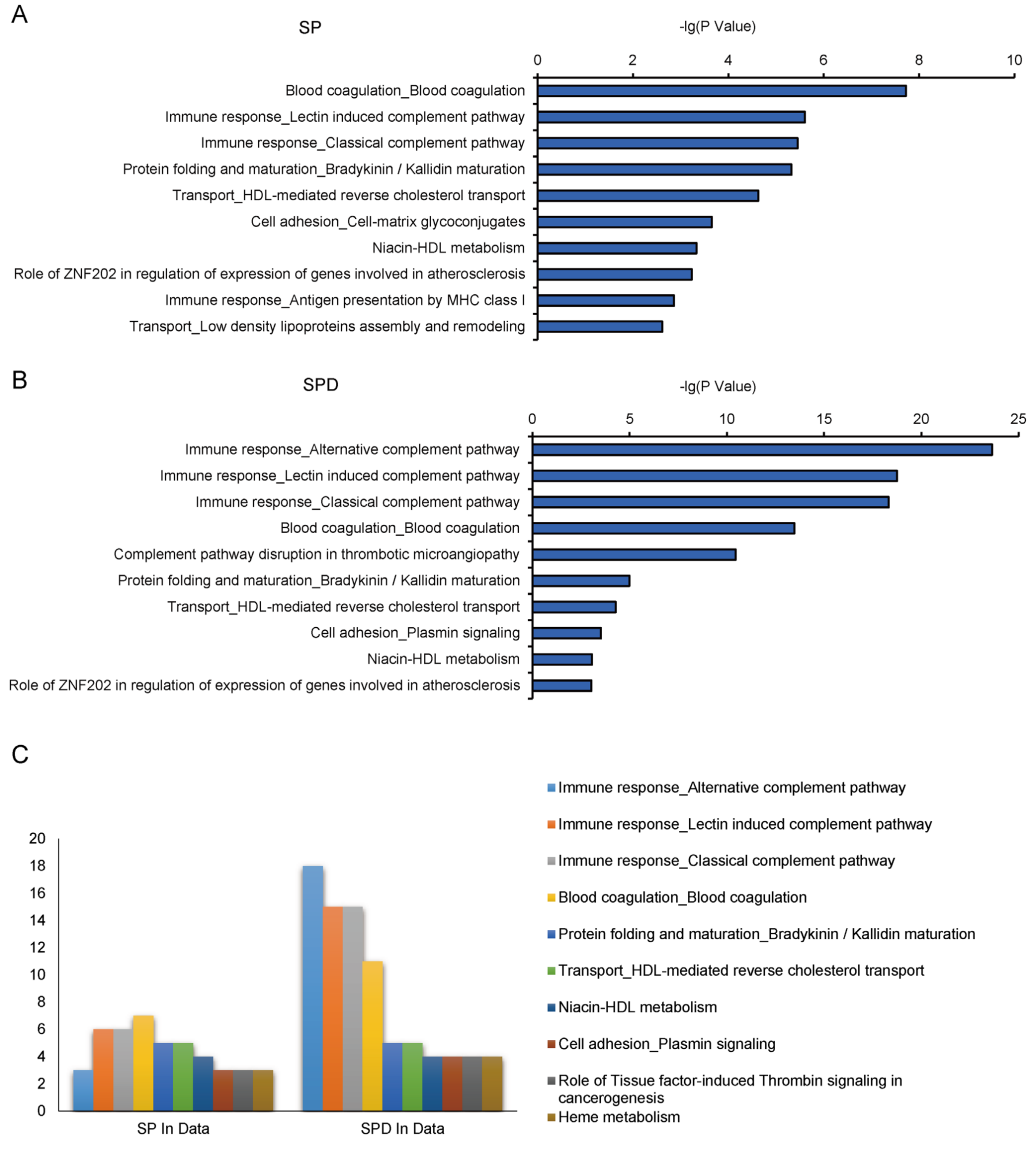
Pathway analysis of 184 SP- or SPD-regulated proteins in aged rat hearts 10 of the top 33 enriched pathways in the SP group **A.** 10 of the top 40 enriched pathways in the SPD group **B.** 10 of the top 24 enriched pathways in both the SP and SPD groups **C.** The number of protein hits in each pathway is shown.

The MetaCore tool was used to study canonical pathways differentially regulated by SP and SPD in aged rat hearts. Thirty-three and 40 pathways were enriched in SP- and SPD-treated rat hearts, respectively (*P* < 0.05) (Supplementary Table 2). Crucial SP-altered biological processes included, “blood coagulation” (*P* = 1.89e^-08^), “immune response, lectin induced complement pathway” (*P* = 2.48e^-06^), and “immune response, classical complement pathway” (*P* = 3.51e^-06^) (Figure 4A). SPD-altered processes included, “immune response, alternative complement pathway” (*P* = 2.30e^-24^), “immune response, lectin induced complement pathway” (*P* = 1.78e^-19^), and “immune response, classical complement pathway” (*P* = 4.84e^-19^) (Figure 4B). Twenty-four of these pathways were altered by both SP and SPD (Figure 4C), including immune response, blood coagulation, protein folding and maturation, HDL-mediated reverse cholesterol transport, and cell adhesion.

**Figure 5:**
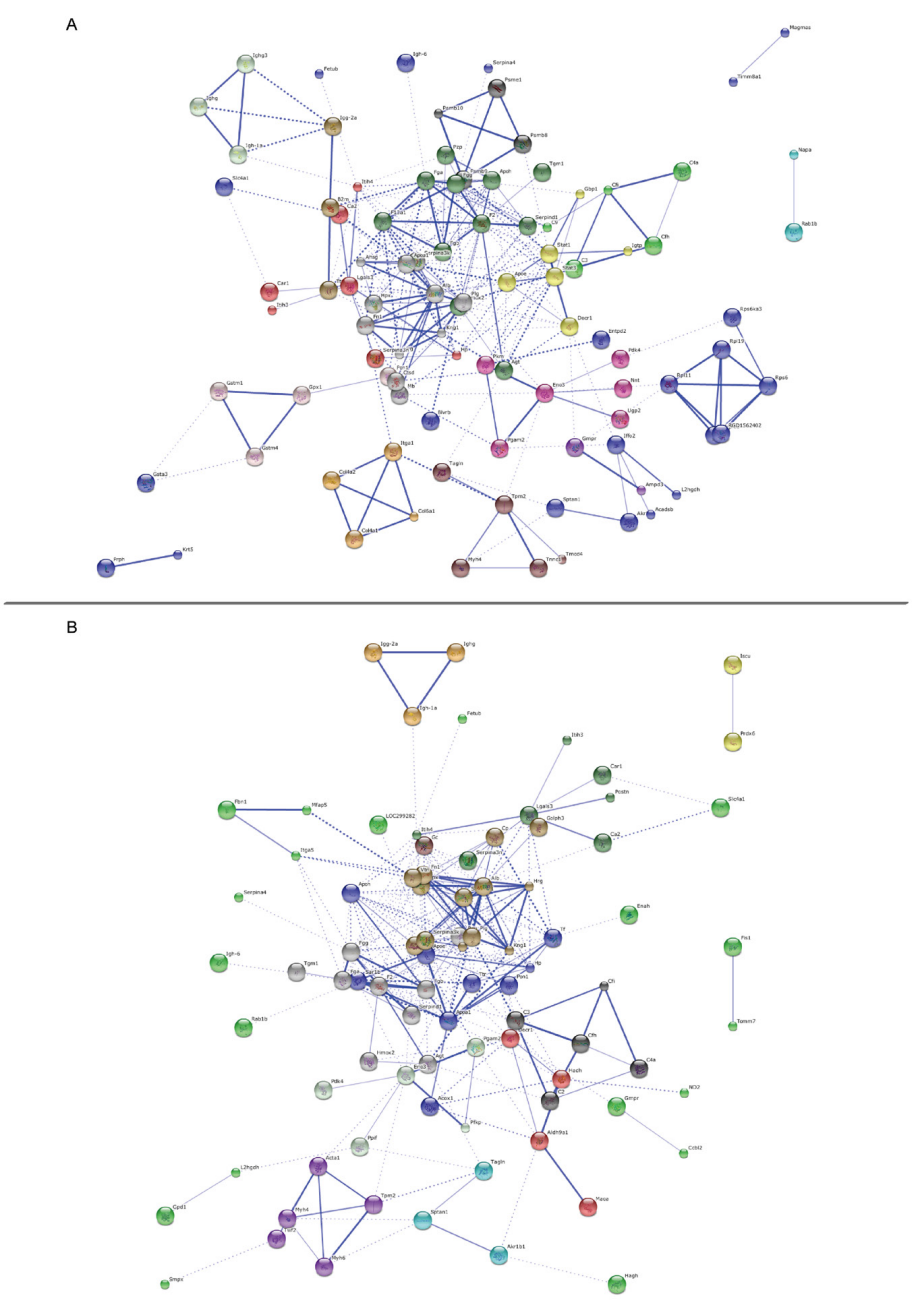
Protein-protein interaction networks Predicted protein-protein interaction network of proteins associated with polyamine treatment in aging rats. Nodes represent proteins and lines indicate protein-protein interactions. Node interiors represent protein structures. Line thickness indicates the grade of evidence for a given interaction. PPI networks for SP-treated **A**. or SPD-treated **B**. *vs.* untreated aging rats.

We then examined protein-protein interactions (PPI) for 131 (STRING annotation 126) proteins differentially expressed following SP treatment and 128 (STRING annotation 122) proteins differentially expressed following SPD treatment compared to the old control group. Proteins belonged to major networks affecting multiple top GO pathways, such as F2, Plg, and Fgg protein networks in SP-treated rats (Figure 5A), and serpina1, F2, and Plg protein networks in SPD-treated rats (Figure 5B).

### Differential metabolic profiles in polyamine-treated aged rat hearts

**Figure 6:**
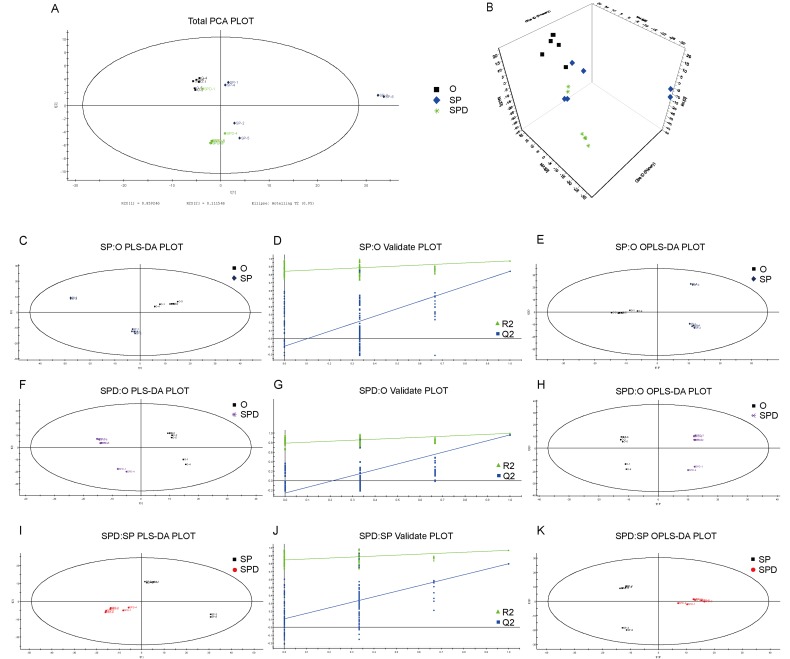
Experimental model robustness and predictive ability Score plot of PCA model with untreated (O), and SP- or SPD-treated aged rat heart tissue **A.** & **B.** Score plot of PLS-DA model with untreated (O), and SP- or SPD-treated aged rat heart tissue **C.**, **F.**, & **I.** Corresponding PLS-DA validation plots. Two hundred permutations were performed, and resulting R2 and Q2 values were plotted **D.**, **G.**, & **J.** Green triangle: R2; blue square: Q2. Score plot of OPLS-DA model with untreated (O), and SP- or SPD-treated aged rat heart tissue **E.**, **H.**, & **K.**

We identified characterized metabolites in senescent heart tissues from SP- or SPD-treated rats using GC-MS-based metabolomics. Six hundred sixty-one peaks were acquired using GC-MS, and 635 metabolites were identified. Based on the full metabolic dataset, PCA analysis identified four principal components. PLS-DA and OPLS-DA score plots (Figure 6A and 6B) separated metabolites from aged, SP- or SPD-treated rat hearts into distinct clusters. PLS-DA revealed distinct metabolic alterations between the groups (SP/O: R2X = 0.549, R2Y = 0.970, and Q2 = 0.842; SPD/O: R2X = 0.491, R2Y = 0.996, and Q2 = 0. 961; SPD/SP: R2X = 0.577, R2Y = 0.967, and Q2 = 0.796) (Figure 6). A validation plot indicated that the PLS-DA model was valid; the Q2 regression line (blue) had a negative intercept and all permuted R2-values (green, left) were lower than the original point of the R2 value (right). OPLS-DA (SP/O: R2Y = 0.970, Q2 = 0.738; SPD/O: R2Y = 0.996, Q2 = 0.904; SPD/SP: R2Y = 0.967, Q2 = 0.732) showed clear differences in cardiac tissue between the groups (Figure 6E, 6H, & 6K).

### Cardiac metabolite screening and identification in polyamine-treated aged rats

In total, 57 (KEGG annotation 38) metabolites were differently regulated in SP-treated rat hearts compared to untreated old rats; 28 were upregulated and 29 were downregulated (Supplementary Table 3). Two hundred (KEGG annotation 118) metabolites were differentially regulated in SPD-treated rat hearts compared to untreated old rats; 24were upregulated and 176 were downregulated (Supplementary Table 4).

**Figure 7:**
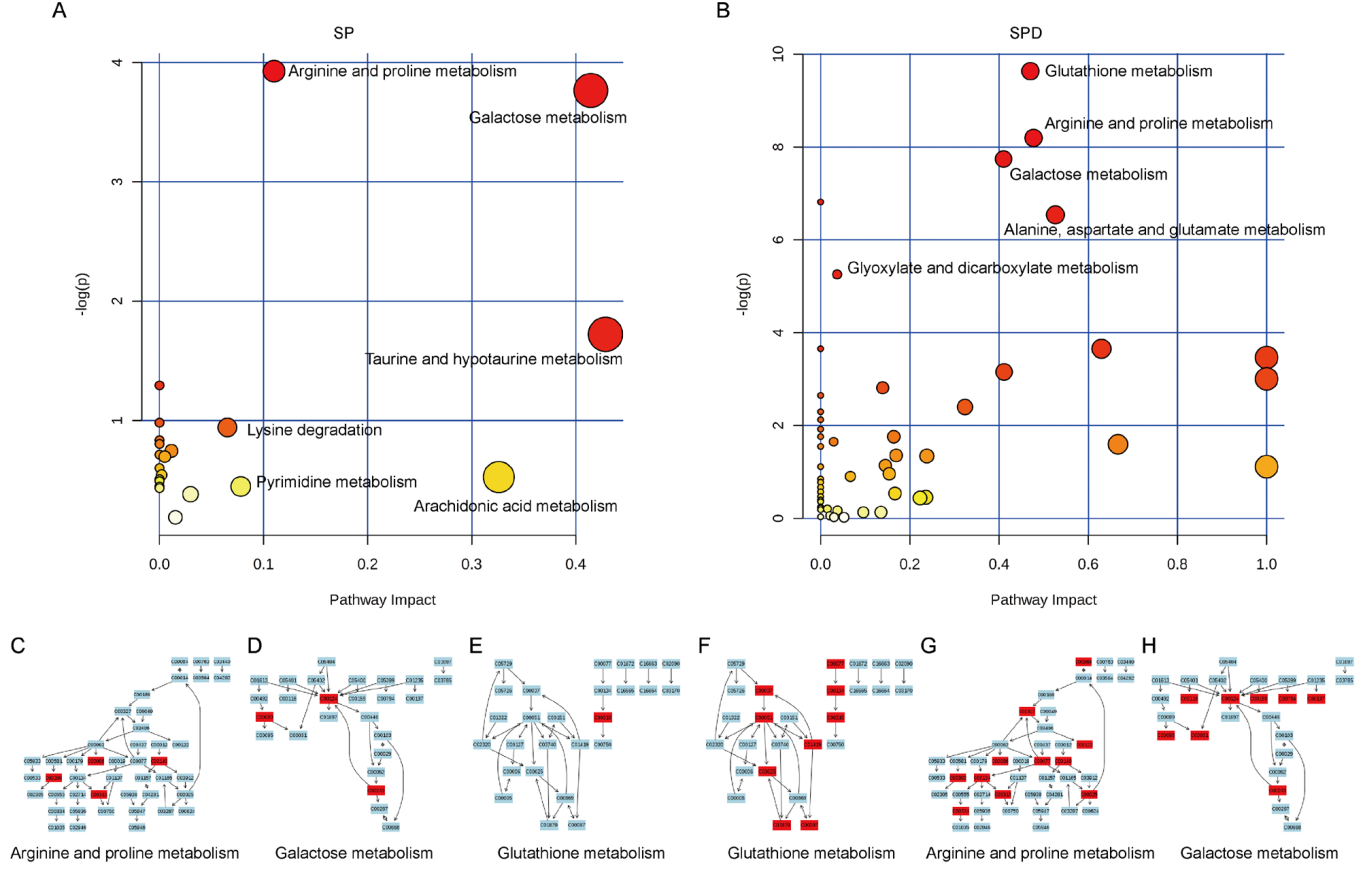
Pathway analysis of aged rat heart metabolites The most relevant pathways were analyzed using the Metaboanalyst. A Google-map style interactive visualization system was applied to facilitate data exploration and generate pathway views. Representative pathway analysis of metabolites in SP- **A.** or SPD-treated **B.** rat senescent heart tissue. SP- **C.**, **D.**, & **E.** or SPD-modulated **F.**, **G.**, & **H.** metabolites (KEGG ID) are shown in red, and are associated with arginine and proline metabolism, galactose metabolism, and glutathione metabolism pathways.

The most relevant pathways were analyzed by pathway enrichment and topology analysis. Metabolites from SP-treated rat hearts were primarily involved in 12 pathways (*P* < 0.05) (Table-3), including arginine and proline metabolism, galactose metabolism, and taurine and hypotaurine metabolism (Figure 7A). Metabolites such as urea, L-proline, creatine, SPD, and taurine were suppressed in SP-treated rat hearts. Metabolites from SPD-treated rat hearts were primarily involved in 11 pathways (*P* < 0.05) (Table-4), including glutathione metabolism, arginine and proline metabolism, and galactose metabolism(Figure 7B). Glycine, glutathione, L-glutamic acid, pyroglutamic acid, L-cysteine, ornithine, putrescine, SPD, cysteinylglycine, citrulline, fumaric acid, L-proline, ornithine, creatine, putrescine, glycerol, D-mannose, myoinositol, D-fructose, D-glucose, alpha-lactose, and gamma-aminobutyric acid levels were suppressed in SPD-treated rat hearts. These metabolites are primarily associated with amino acid, carbohydrate, and lipid metabolism.

**Table 3:** Analyzed pathways of metabolomics data differently regulated in heart tussue of SP treatment rats using MetaboAnalyst 3.0

Term	Total	Expected	Hits	Raw *p*
Arginine and proline metabolism	44	1.067	4	0.019698
Galactose metabolism	26	0.63053	3	0.023149
Taurine and hypotaurine metabolism	8	0.19401	1	0.17873
Nicotinate and nicotinamide metabolism	13	0.31526	1	0.27424
Pentose phosphate pathway	19	0.46077	1	0.37469
beta-Alanine metabolism	19	0.46077	1	0.37469
Lysine degradation	20	0.48502	1	0.39006
Starch and sucrose metabolism	23	0.55777	1	0.43401
Alanine, aspartate and glutamate metabolism	24	0.58203	1	0.44797
Glutathione metabolism	26	0.63053	1	0.47488
Aminoacyl-tRNA biosynthesis	67	1.6248	2	0.49027
Purine metabolism	68	1.6491	2	0.49829

**Table 4:** Analyzed pathways of metabolomics data differently regulated in heart tussue of SPD treatment rats using MetaboAnalyst 3.0

Term	Total	Expected	Hits	Raw p
Glutathione metabolism	26	1.9843	9	6.56E-05
Arginine and proline metabolism	44	3.3581	11	0.000276
Galactose metabolism	26	1.9843	8	0.000435
Aminoacyl-tRNA biosynthesis	67	5.1134	13	0.001097
Alanine, aspartate and glutamate metabolism	24	1.8317	7	0.001449
Glyoxylate and dicarboxylate metabolism	16	1.2211	5	0.005219
Nitrogen metabolism	9	0.68688	3	0.025863
Phenylalanine metabolism	9	0.68688	3	0.025863
Phenylalanine, tyrosine and tryptophan biosynthesis	4	0.30528	2	0.031278
Glycerolipid metabolism	18	1.3738	4	0.042615
D-Glutamine and D-glutamate metabolism	5	0.3816	2	0.049555

### Integrated analysis of polyamine-modulated cardiac proteins and metabolites in aged rats

**Figure 8:**
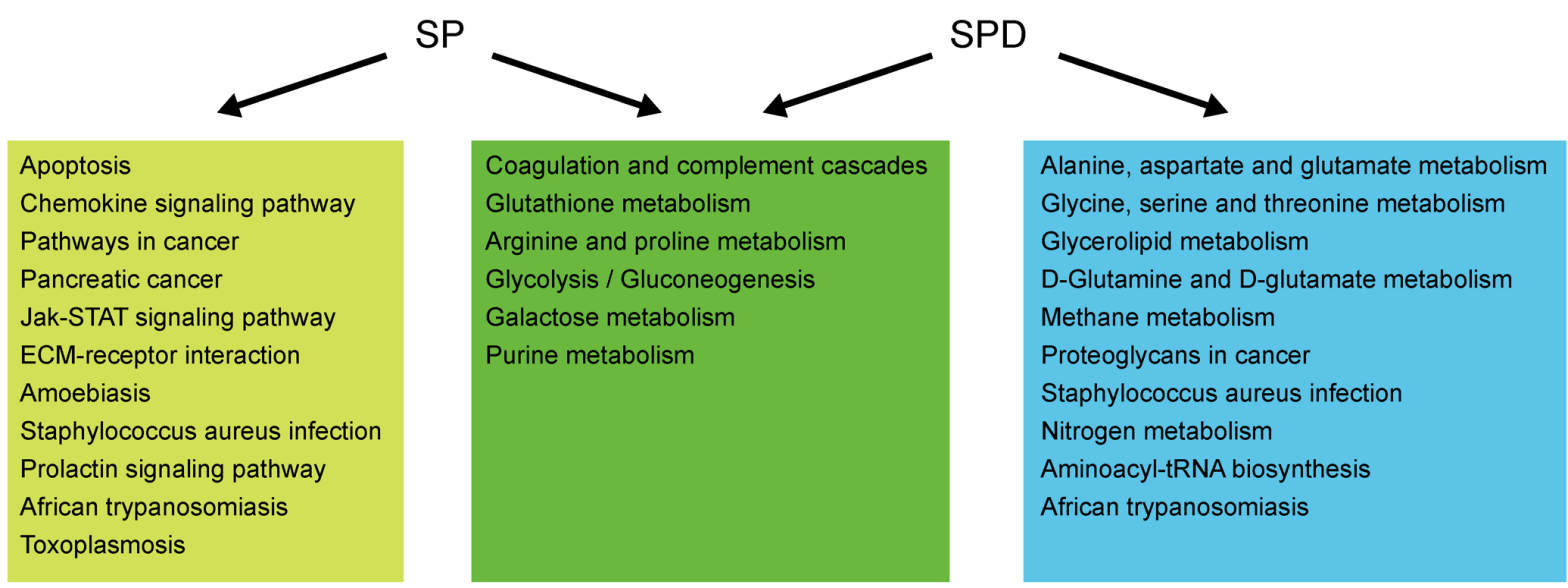
Pathway analysis of proteins and metabolites differentially expressed/produced following SP or SPD treatment Pathways altered in both the SP and SPD groups are shown in green boxes. Pathways altered only in the SP or SPD group are shown in yellow or blue boxes, respectively.

**Figure 9:**
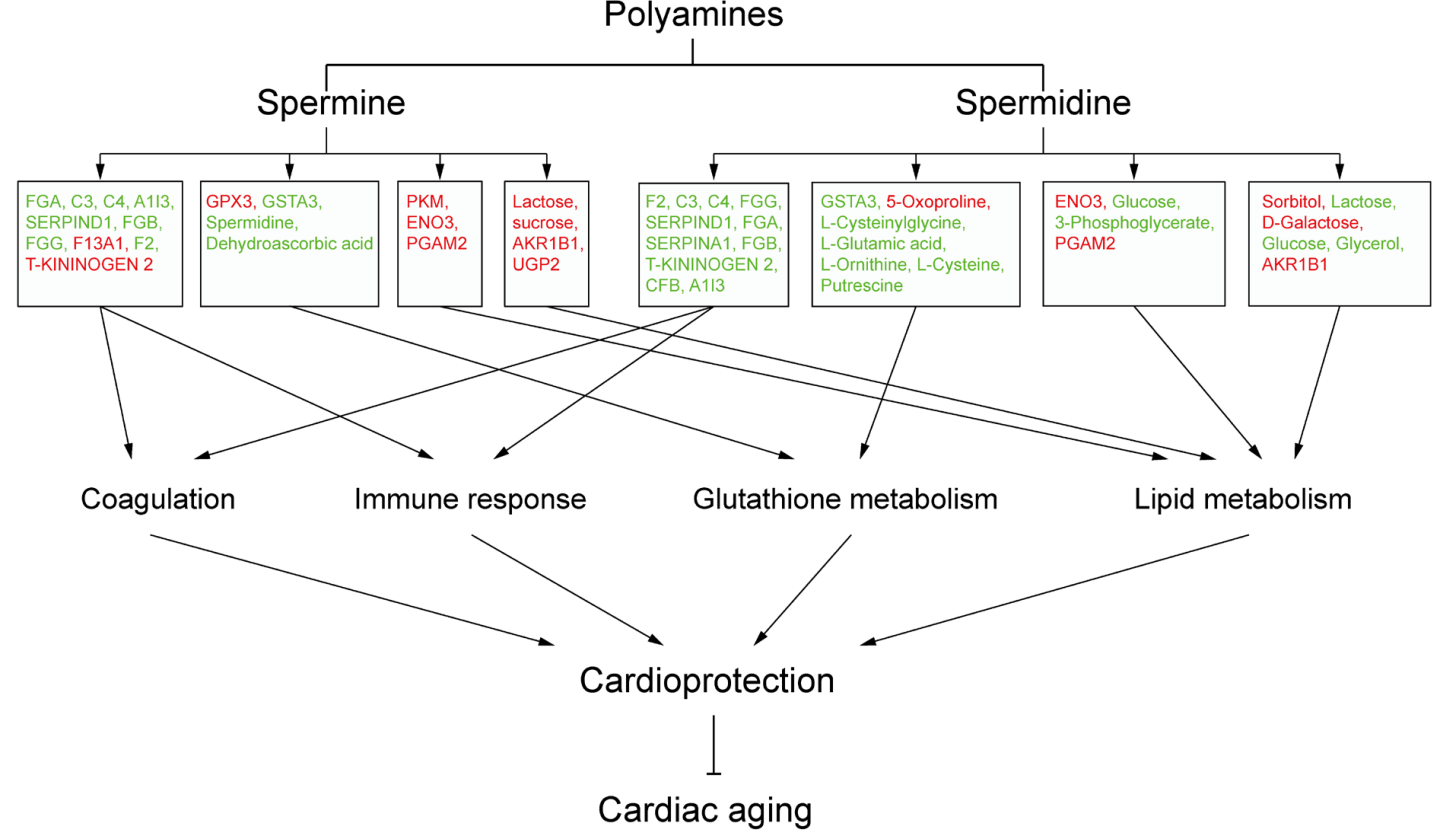
Schematic overview of polyamine-related cardioprotective pathways in aged rat hearts Molecules in red are upregulated, and those in green are downregulated.

To investigate whether differentially expressed/produced proteins and metabolites interacted, a KEGG-based network was generated to elucidate SP- and SPD-modulated signaling pathways. Proteins/metabolites that shared interactive relations with differentially expressed proteins and metabolites identified in this study were added to the set. The hypergeometric test was performed for pathway enrichment analysis, and pathways with *P* < 0.05 were filtered (Supplementary Table 5-6). Six pathways (Figure 8) were altered in both SP- and SPD-treated rats, including coagulation and complement cascades (ko04610), arginine and proline metabolism (ko00330), glycolysis/gluconeogenesis (ko00010), galactose metabolism (ko00052), and glutathione metabolism (ko00480). Pathways altered only by SP involved apoptosis (ko04210) and cancer (ko05200 and ko05212), while SPD treatment affected amino acid metabolism (ko00250 and ko00260) and glycerolipid metabolism (ko00561). F2, C3, and C4 were identified as key coagulation and complement cascade regulators. Coagulation, immune response, lipid metabolism, and glutathione metabolism are associated with cardioprotection and anti-aging in the heart (Figure 9).

## DISCUSSION

Age-induced cardiomyopathy in the mammalian heart is characterized by myocardial hypertrophy, fibrosis, and a predisposition towards cardiomyocyte apoptosis. Interstitial fibrosis of the atria, sinoatrial node (SAN), and ventricles adversely affects myocardial function in older adults [24]. Increased cardiomyocyte apoptosis leads to myocardial damage and eventually heart failure in aging C57BL/6 male mice [25]. Thus, preventing fibrosis and apoptosis are important strategies to prevent heart disease [26]. This study showed that six weeks of SP or SPD treatment reversed previously existing age-associated myocardial morphology changes and myocardial fibrosis, and inhibited cell apoptosis in aging rat hearts. SP and SPD both modulated rat heart proteins and metabolites associated with immune response, blood coagulation, lipid metabolism, and glucose metabolism. However, SP was more associated with apoptosis, chemokine signaling, and cancer-related pathways, while SPD was associated with amino acid and glycerolipid metabolism.

Blood coagulation proteins regulate early atherosclerosis and its progression [27-30]. Otto, *et al.* reported that aortic sclerosis increases risk of myocardial infarction and cardiovascular-associated death in the elderly [31]. Our proteomic analysis showed that SP and SPD regulate coagulation-related protein expression. Protein-protein interaction analyses revealed that F2 and serpina1 interact within smaller networking groups. F2 is a prothrombin precursor involved in blood homeostasis, inflammation, and wound healing. Aging and cardiovascular risk factors increase plasma levels of hemostatic molecular markers like F2 in the elderly [32]. We found that F2 (SP/O: 0.710 and SPD/O: 0.704) was downregulated in SP- and SPD-treated aging rat hearts. Serpina1 encodes alpha-1 antitrypsin (AAT), an acute-phase inflammation marker synthesized predominantly in hepatocytes that inhibits proteases, such as elastase, trypsin, thrombin, and bacterial proteases. Serpina1 upregulation is associated with multiple CVDs, including heart failure [33-35], and levels were lower in non-ischemic right ventricles (RV) than in ischemic RVs. Our proteomic analysis showed that serpina1 is downregulated (SPD/O: 0.650) in SPD-treated aging rat hearts. We speculate that SP and/or SPD may reduce morbidity and mortality from myocardial infarction and CVD by inhibiting aortic sclerosis.

Increased immune activity can lead to chronic inflammation and degenerative diseases. The protein and mRNA expression levels of complement C3 were found to be associated with the occurrence and development of coronary heart disease [36], higher C3 was significantly associated with presence and greater extent of arterial calcification, and C3 could be a potential non-invasive biomarker of early diagnosis of atherosclerosis [37]. Activation of the immune response by elevating hydroperoxides levels, further activating NF-kB signaling, may be a key aging feature in the mouse heart [38]. In addition, Lian et al reported that astroglial NF-kB/C3 activation led to impaired synaptic density and dendritic morphology in brain tissue of Alzheimer’s disease (AD) patients [39], and the expressions of C3, C4 and C5 mRNA increased with age in C57BL/6 mice brain [37-39]. Here, our proteomic analysis revealed decreased C3 (SP/O: 0.811 and SPD/O: 0.555) and C4 (SP/O: 0.781 and SPD/O: 0.653) expressions in both SP- and SPD-treated rats, which may contribute to the anti-aging effects of these polyamines.

Fatty acids supply 50-75% of the energy to the normal heart, while glucose oxidation and glycolysis provide adenosine triphosphate (ATP) [40]. Under normal conditions, glucose metabolism cooperates with fatty acid metabolism to produce ATP. However, physiological (aging) and pathophysiological (hypertension and diabetes) conditions can alter glucose and fatty acid metabolism, increasing risk of hypertension, insulin resistance, abnormal cholesterol and possible heart disease. During cardiac aging, increased generation of reactive oxygen species (ROS) can damage mitochondria and lead to perturb fuel utilization. The fatty acid β-oxidation impaired has been observed in aged mice heart [41, 42]; the amount of total fatty acid concentrations decreased and the arachidonic acid concentrations increased in aged rat hearts [43]. Reverse in the age-related reduction in fatty acid oxidation improves mitochondrial metabolism and energetic profile in old C57BL/6 mice hearts [44]; decreasing arachidonic acid metabolism has neuroprotective role in neurocognitive disorder patients and rat of neuroinflammation [45]. On the other hand, aging individuals are more likely to suffer from lower glucose levels than younger individuals, as the age increases, glucose tolerance becomes worse and glucose transporters decrease in rat [46]. Galactose is readily converted to glucose, galactose at 15% of daily intake improves hepatic insulin sensitivity in rats; galactose can also improve glucose metabolism by enhancing mitochondrial oxidative phosphorylation in cultured human myotubes [47]. While stimulating mitochondrial glucose oxidation has been suggested as a viable therapeutic strategy to compensate for the energetically ‘starved’ heart [48]. Our study showed that SP and SPD reversed lipid and glucose metabolism changes induced by aging in the heart, including glycerol (SPD/O: 0.60), arachidonic acid (SP/O: 0.360), and galactose (SP/O: 3.920 and SPD/O: 2.810) levels. Therefore, we speculate that SP and SPD can inhibit metabolic remodeling and decrease age-related CVD risk.

Glutathione is a major anti-oxidant in cells [49], and reductions may result from increased ROS concentrations in liver tissues [50]. Our metabolomic analysis showed that SPD treatment suppressed glutathione metabolism-related metabolites, including glycine, glutathione, L-glutamic acid, pyroglutamic acid, L-cysteine, ornithine, putrescine, SPD, and cysteinylglycine. Decreased glutathione may in turn lead to increased ROS concentrations, possibly indicating increased mitochondrial β-oxidation. This may lead to more robust fat combustion, similar to a “fasted” metabolic state [35], potentially depleting lipids and inhibiting dyslipidemia in aged rat hearts.

Proteomics methods analyze the entire expressed protein profile of a biological sample [19], while metabolomics provides a metabolite fingerprint [23]. Together, proteomic and metabolomic analyses can provide comprehensive mechanistic information about diverse pathologies [51, 52]. We integrated proteomic and metabolomic data streams to elucidate mechanisms underlying the anti-aging effects of SP and SPD in the heart. However, our study had limitations. Natural deaths in the 22-24-month-old rats reduced group sample sizes from more than six to only three. Additionally, proteomics technologies have expanded; strong cation exchange-reverse phase (SCX-RP) 2D LC-MS-MS applied to a soluble protein lysate from mouse embryonic fibroblast cells identified more than 5000 proteins [53]. This technique could identify more proteins more accurately in future studies.

In summary, using an integrated proteomics and metabolomics approach, we identified differentially expressed proteins and metabolites in aging rat hearts following polyamine treatment. We systematically analyzed the functions of these proteins and metabolites, and found that exogenous SP and SPD exerted anti-aging effects in rat hearts likely by regulating immune response, blood coagulation, lipid metabolism, and glutathione metabolism pathways. Future studies will assess whether polyamines work against heart aging through F2 and glutathione. Our study provides novel molecular information on the anti-aging effects of polyamines in the rat heart, and supports SP and SPD as potential clinical therapeutics targeting heart disease.

## MATERIALS AND METHODS

### Animal model and treatment protocol

Three- and 22-24-month-old male Wistar rats were obtained from the Animal Center of Harbin Medical University. Three-month-old male rats were considered the young control group (Y) (*n* = 3), and 22-24-month-old male rats were randomly assigned to the old control (O) (*n* = 3), SP (*n* = 3), and SPD (*n* = 3) groups. SP and SPD group rats were treated with SP (2.5 mg/kg/day) or SPD (10 mg/kg/day) intraperitoneally for 6 weeks. SP and SPD were obtained from Sigma Chemical Co. (St. Louis, MO, USA). Rats were treated according to the Guide for Care and Use of Laboratory Animals published by the China National Institutes of Health. All rats were housed under environmental conditions of constant temperature and humidity, with free access to standard rodent chow and water.

### Histological and morphological analyses

Left ventricular tissues were excised and fixed in 4% paraformaldehyde at room temperature for >1 d, and then prepared for H&E staining, Masson staining, and cell apoptosis assay. Tissues were embedded in paraffin, sectioned at 5-μm thickness, and stained with Mayer’s hematoxylin followed by 1% eosin alcohol solution, Masson’s trichrome, and PicroSirius red. Stained sections were visualized at 40× magnification, and volume fraction of collagen (VFC) fibril structures were quantified from three sections per heart. Results were averaged from five high power random fields from each section. A terminal deoxynucleotidyl transferase mediated dUTP nick end-labeling (TUNEL) assay was applied to detect apoptotic myocytes using the Cell Death Detection Kit (Roche, Basel, Switzerland) following the manufacturer’s instructions. Deparaffinized and rehydrated heart sections were incubated with TUNEL reaction mixture, including terminal deoxynucleotidyl transferase (TdT) and fluorescein-dUTP after permeabilization. Sections were developed using diaminobenzidine (DAB; Sigma Chemical Co.; St. Louis, MO, USA), and nuclei were counterstained using hematoxylin. Percentage of apoptotic cells was calculated from three sections per block. One hundred cells were counted in each of four section fields, which were randomly chosen at 200× magnification.

### iTRAQ proteomics analysis

Details of protein sample preparation, tryptic digestion, and proteomic profiling acquisition are provided in Supporting Information. GO analysis was performed with iTRAQ results using the open access DAVID bioinformatics platform (Database for Annotation, Visualization and Integrated Discovery, http://www.david.abcc.ncifcrf.gov website). DAVID was used to identify enriched biological processes related to differentially expressed proteins identified via iTRAQ analysis. Pathway analysis was performed with differentially expressed proteins using MetaCore software. The following criteria were used to determine differential protein expression in SP- or SPD-treated rat hearts compared with controls (O group): *P* < 0.05, fold change >1.2 (or < 0.833), and at least two unique peptides.

### Protein-protein interaction analysis

Co-expressed genes may be (1) controlled by the same transcriptional regulatory program, (2) functionally related, or (3) members of the same pathway or protein complex. Protein-protein network analysis was performed for differentially expressed proteins identified via iTRAQ analysis using STRING 10.0 (http://www.string-db.org/), an online database of known and predicted protein-protein interactions, based on two types of evidence: experimental (protein-protein interaction databases) and text-mining (scientific literature abstracts). Differently expressed proteins were mapped to the STRING database and known and predicted associations were scored and integrated, with a combined-score threshold of >0.4. Differentially expressed proteins were visualized after KMEANS clustering. The interaction network was constructed by integrating these relationships.

### Metabolomic data analysis

Sample preparation, metabolic profiling acquisition, data processing, and quality control details are provided in Supporting Information. Chroma TOF 4.3X software (LECO Corporation, St Joseph, MI, USA) and the LECO-Fiehn Rtx5 database were used to process GC/MS data for extraction of raw peaks, data baseline filtering and calibration, peak alignment, deconvolution analysis, peak identification, and peak area integration.

Missing raw data values were filled in using half the minimum value, interquartile range denoising was performed to detect peaks and filter metabolites, and data were normalized to an internal standard. Peak numbers, sample names, and normalized peak areas were analyzed using the SIMCA-P 11.5 software package (Umetrics, Umea, Sweden) for principal component analysis (PCA), partial least squares discriminant analysis (PLS-DA), and orthogonal projections to latent structures-discriminant analysis (OPLS-DA). PCA showed the distribution of the original data. To better separate groups and understand variables responsible for classifications, a supervised PLS-DA was applied. Based on an OPLS-DA, a loading plot was constructed showing the contributions of variables to differences between two groups.

To refine this analysis, the first principal component of variable importance projection (VIP) was obtained. VIP values >1.0 were selected as differentially produced metabolites. The remaining variables were assessed by Student’s *t*-test, *P* < 0.05. Variables were discarded between two comparison groups. Additionally, commercial databases, including KEGG (http://www.genome.jp/kegg/) and NIST (http://www.nist.gov/index.html), were used to search for metabolites.

Pathway analysis of potential metabolites (including pathway enrichment analysis, pathway topology analysis, and visualization) was performed in MetaboAnalyst 3.0 to identify the top altered pathways. Other implicated pathways were explored using databases and the peer-reviewed literature.

### Pathway analysis of differentially expressed proteins and metabolites

KGML files that indicate how proteins (boxes) are linked by “relations” and how chemical compounds (circles) are linked by “reactions” were downloaded from KEGG. Interactive relations for proteins and metabolites in this study, including protein-protein relation (PPrel), protein-compound relation (PCrel), and protein-compound in a single reaction, are based on KEGG information. The hypergeometric test was performed for pathway enrichment analysis.

### Statistical analysis

All statistical analyses were performed using SPSS 17.0 (SPSS. Chicago, IL, USA). Values are showed as means ± standard deviation (SD). Differences were considered significant at *P* < 0.05.

## SUPPLEMENTARY MATERIALS FIGURE AND TABLES




