# Supplementary material for: Spermine and spermidine reversed age-related cardiac deterioration in rats
Source: Oncotarget. 2017 May 31;8(39):64793–808. doi: 10.18632/oncotarget.18334 (PMC5630292; doi:10.18632/oncotarget.18334)
Supplement: Supplementary file 1 [file oncotarget-08-64793-s001.pdf]

Spermine and spermidine reversed age-related cardiac deterioration in rats

Supplementary Material

MATERIALS AND METHODS

iTRAQ quantitative proteomics analysis.

Preparation of crude tissue extracts

For each group, appropriate amount of the left ventricle tissue was treated with SDT buffer (4% SDS, 1mM DTT, 150mM TrisHCl pH8.0), and homogenized individually using a Tenbroeck Tissue Grinders (Wheaton). The homogenate of each left ventricle was boiling in water bath at 100 °C for 5 minutes. A sonicator set at power setting at 80 watts was used to disrupt cell membranes. Every sample was sonicated with ten 10-second pulses with 15 seconds break between pulses. The individual samples were centrifuged at 14,000 rpm at 4 °C for 30 minutes on a refrigerated ultracentrifuge (Eppendorf5430R) to remove cell debris after boiled again in water bath at 100 °C for 5 minutes. The supernatant was assembled individually, the bicinchoninic (BCA) protein determination concentration kit was used to determine total concentrations.

Protein Digestion and iTRAQ labeling

Every aged rat ventricle preparation was evaluated individually, where a 400 µg aliquot of protein was placed in an individual eppendorf tube, and was incorporated into 200 µl UA buffer (8 M Urea, 150 mM Tris-HCl pH 8.0) for removing the detergent, DTT and other low-molecular-weight components, by repeated ultrafiltration (Microcon units, 30 kD). Then the samples were incubated for 20 minutes in darkness after 100 µl 0.05 M iodoacetamide in UA buffer was added to dispute reduced decreased cysteine residues. The filters were washed with 100 µl UA buffer three times before washed with 100µl DS buffer (50 mM triethylammoniumbicarbonate at pH 8.5) twice. After all, 2 µg trypsin (Promega) in 40 µl DS buffer was used to digested the protein suspensions overnight at 37 °C, and the giving peptides were gathered as a colature. The peptide content was evaluated by UV light spectral density at 280 nm using an extinctions coefficient of 1.1 of 0.1% (g/l) solution, which was calculated on the basis of the frequency of tyrosine and tryptophan in vertebrate proteins.

Each iTRAQ experiment (3 samples) was then taken on to the iTRAQ labeling protocol. 54ug of each sample was treated individually with the iTRAQ labeling reagent. With iTRAQ labeling performed on the control pool by applying the iTRAQ labeling reagent 114, both perparations of PSP and PSPD were treated individually with iTRAQ tagging reagents 116 and 117, respectively. The samples were then multiplexed and vacuum dried.

Protein fractionation

Peptide Fractionation with Strong Cation Exchange (SCX) Chromatography

The fractionation of iTRAQ labeled peptides was performed using SCX chromatography over the AKTA purifier system (GE healthcare). The dried peptide mixture was reconstituted and acidified with 2 ml buffer A (10 mM KH2PO4 in 25% of ACN, pH 2.7) and loaded onto a polysulfoethyl 4.6 x 100 mm column (5 µm, 200 Å, PolyLC Inc, Maryland, U.S.A.).

The peptides were eluted at a flow rate of 1 ml/min with a gradient of 0%–10% buffer B (500 mM KCl, 10 mM KH2PO4 in 25% of ACN, pH 2.7) for 2 min, 10–20% buffer B for 25 min, 20%–45% buffer B for 5 min, and 50%–100% buffer B for 5 min. The elution was monitored by absorbance at 214 nm, and fractions were collected every 1 min. The collected fractions (about 30 fractions) were finally combined into 4 pools and desalted on C18 Cartridges (Empore™ SPE Cartridges C18 (standard density), bed I.D. 7 mm, volume 3 ml, Sigma). Each fraction was concentrated by vacuum centrifugation and reconstituted in 40 µl of 0.1% (v/v) trifluoroacetic acid. All samples were stored at -80°C until LC-MS/MS analysis.

Liquid Chromatography (LC) - Electrospray Ionization (ESI) Tandem MS (MS/MS) Analysis by Q Exactive.

Experiments were performed on a Q Exactive mass spectrometer that was coupled to Easy nLC (Proxeon Biosystems, now Thermo Fisher Scientific). 10 µl of each fraction was injected for nanoLC-MS/MS analysis. The peptide mixture (5 µg) was loaded onto a the C18-reversed phase column (15 cm long, 75 µm inner diameter) packed in-house with RP-C18 5µm resin in buffer A (0.1% Formic acid) and separated with a linear gradient of buffer B (80% acetonitrile and 0.1% Formic acid) at a flow rate of 250 nl/min controlled by IntelliFlow technology over 140 min. MS data was acquired using a data-dependent top10 method dynamically choosing the most abundant precursor ions from the survey scan (300–1800 m/z) for HCD fragmentation. Determination of the target value is based on predictive Automatic Gain Control (pAGC). Dynamic exclusion duration was 60 s. Survey scans were acquired at a resolution of 70,000 at m/z 200 and resolution for HCD spectra was set to 17,500 at m/z 200. Normalized collision energy was 30 eV and the underfill ratio, which specifies the minimum percentage of the target value likely to be reached at maximum fill time, was defined as 0.1%. The instrument was run with peptide recognition mode enabled.

Metabolomics analysis

Tissue sample pretreatment

GC-MS analysis was performed to quantify the metabolites concentrations of rat heart tissue samples. 20 mg rat heart tissue sample was thawed at room temperature, subsequently, was transferred into a 1.5 mL centrifuge tube with 1.0 mL mixture solution, which including 700µL of 75% methanol and 100µL chloroform. After these 100µL L-2-chlorophenylalanine was added as an internal quantitative standard. 60 min of Sonicate was used to separate the mixture into extraction of metabolites and precipitation of proteins. The components without the precipitated proteins were remaining in the solution, after centrifuged at 12,000 g for 10 min. To concentrate and obtain the metabolites in the solution, 600µL supernatant was transferred to a glass vial (4 mL) with PTEE-lined screw cap, and dried in a vacuum concentrator at 50 ℃. After the sample dried, 75 µL BSTFA (containing 1% TMCS) was added in the vial, the derivatization reaction was conducted under 400 Watt microwave for 10 min. Finally, the sample was under derivatization and cooled to room temperature, 1µL of which was injected in the GC-MS for analysis.

GC-MS

A Pegasus 4D GC×GC-TOF mass spectrometer (LECO Chroma TOF PEGASUS 4D, LECO, UDA) was used for sample analysis. The system was installed with a DB-5MS capillary column, which coated with 95% dimethylpolysiloxane cross-linked with 5% diphenyl. A 1µL aliquot was injected at a split less mode with helium as the carrier gas and the gas flow rate of 1mL/min; the front entry purge flow rate was 3 mL/min. The temperature for injection, transfer line, and ion source temperatures were 250, 240, and 230 ℃, respectively. The initial temperature of the column was first kept at 40 ℃ for 120 sec, then increased to 180 ℃ at the rate of 10℃/min, to 280 ℃ at a rate of 8 ℃/min, where it was maintained for 180 sec. The MS data was acquired in full scan mode with a mass-to-charge ratio (m/z) range of 45-800 at a rate of 100 spectra/s after a solvent delay of 180 sec.

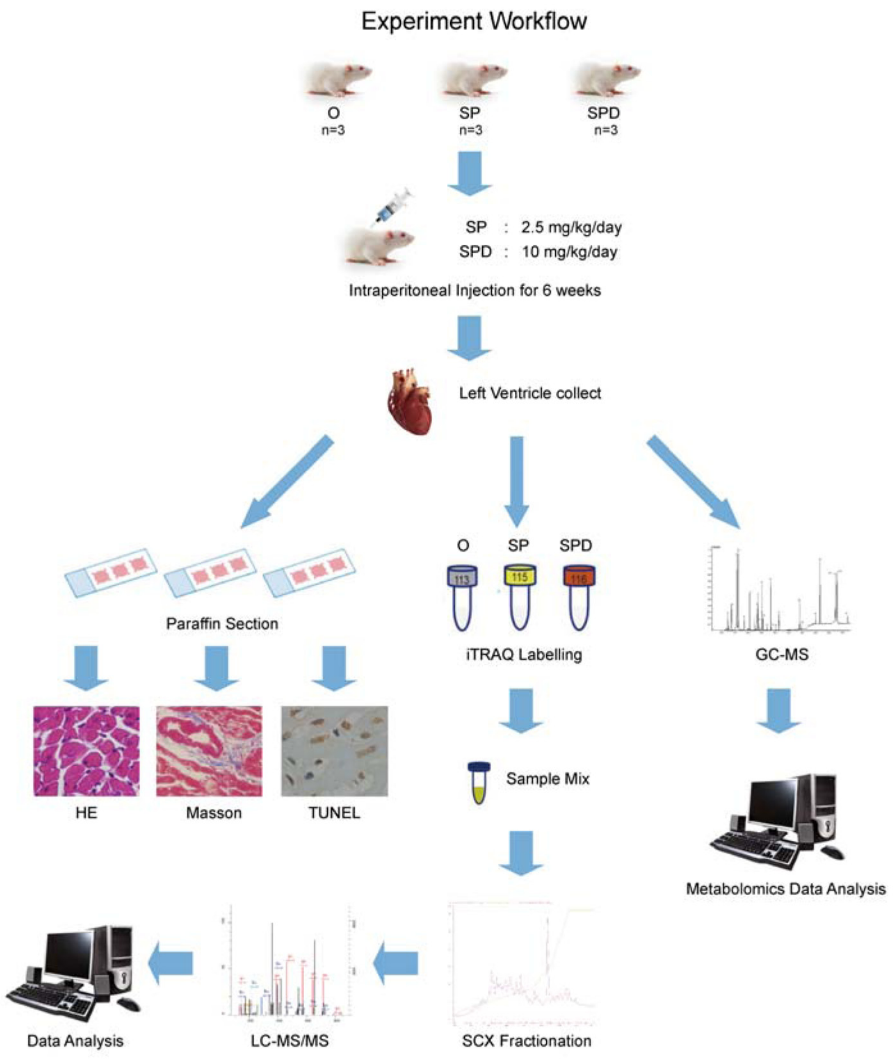

Supplementary Figure 1: Experiment workflow for the present study.

Supplementary Tables see in Supplementary Files
