# Supplementary material for: Spermine and spermidine reversed age-related cardiac deterioration in rats
Source: Oncotarget. 2017 May 31;8(39):64793–808. doi: 10.18632/oncotarget.18334 (PMC5630292; doi:10.18632/oncotarget.18334)
Supplement: Supplementary file 2 [file oncotarget-08-64793-s002.doc]

**Table S-1.** Differentially expressed proteins in rat heart in SP and SPD groups compared with old group.

| No. | Protein name | Gene name | Unique Peptides | SP/O  Fold change | SP/O p value | SPD/O  Fold change | SPD/O p value |
| --- | --- | --- | --- | --- | --- | --- | --- |
|  | Fga protein | A1L114 | 12 | 0.641667598 | 2.53E-05 | 0.512085826 | 3.40E-10 |
|  | Protein tyrosine kinase 9-like (A6-related protein) (Predicted), isoform CRA_b | B0BMY7 | 5 | 0.979599036 | 0.843964 | 1.203777112 | 0.0394016 |
|  | Carbonic anhydrase 1 | CAH1 | 4 | 0.258461284 | 9.40E-38 | 0.385897646 | 4.18E-19 |
|  | Maoa protein (Fragment) | B2GV33 | 13 | 0.838434074 | 0.0941786 | 0.797160048 | 0.0331444 |
|  | Igtp protein | B2GV84 | 3 | 2.0039839 | 1.39E-16 | 1.074355099 | 0.427761 |
|  | Iron-sulfur cluster scaffold homolog (E. coli) | B2RZ79 | 3 | 0.921010008 | 0.434107 | 0.811328792 | 0.0493964 |
|  | Aldh4a1 protein (Fragment) | B4F768 | 11 | 1.212025105 | 0.0222832 | 1.192221814 | 0.0508753 |
|  | Biliverdin reductase B (Flavin reductase (NADPH)) | B5DF65 | 5 | 0.76061366 | 0.00936823 | 0.877511222 | 0.218711 |
|  | Alpha II spectrin | C9EH87 | 2 | 1.315069673 | 0.00113008 | 1.208714483 | 0.0352426 |
|  | Protein Rps6ka3 | D3Z8E0 | 2 | 0.799558858 | 0.0336394 | 0.826845467 | 0.0738501 |
|  | Protein Fundc2 | D3ZAQ0 | 2 | 0.710478249 | 0.00117242 | 0.773794811 | 0.0159858 |
|  | Protein Ttc15 | D3ZE49 | 2 | 0.811711056 | 0.0475693 | 0.845603426 | 0.114744 |
|  | Protein RGD1562402 | D3ZF07 | 2 | 0.72150467 | 0.00193956 | 0.808578816 | 0.0458267 |
|  | Protein Itih4 | D3ZFC6 | 13 | 0.655271636 | 5.99E-05 | 0.552930296 | 2.70E-08 |
|  | RING finger protein 114 | D3ZG22 | 2 | 1.218626487 | 0.018768 | 1.091378417 | 0.332976 |
|  | Protein Postn | D3ZG30 | 2 | 0.83408161 | 0.0848604 | 0.811031052 | 0.0489996 |
|  | Uncharacterized protein | D3ZIX5 | 3 | 0.678993141 | 0.000237173 | 0.636619622 | 2.25E-05 |
|  | Microfibrillar associated protein 5 (Predicted), isoform CRA_a | D3ZJB1 | 2 | 1.273417854 | 0.00406368 | 1.349281482 | 0.000865454 |
|  | Voltage-dependent calcium channel subunit alpha-2/delta-1 | D3ZKP9 | 2 | 1.046328952 | 0.591177 | 1.281983655 | 0.00576111 |
|  | Protein Gbp1 | D3ZKU6 | 8 | 2.268088272 | 2.07E-22 | 0.987255886 | 0.900396 |
|  | Band 4.1-like protein 1 | D3ZLZ8 | 2 | 1.131822973 | 0.141223 | 1.26153141 | 0.00982454 |
|  | Protein Fbn1 | D3ZQM5 | 13 | 0.975643426 | 0.814031 | 1.254094324 | 0.0118667 |
|  | Uncharacterized protein | D3ZSC9 | 2 | 0.662455425 | 9.24E-05 | 0.607614695 | 2.93E-06 |
|  | Protein Tmod4 | D3ZSG3 | 2 | 0.796469269 | 0.0306865 | 1.093463463 | 0.322469 |
|  | Myotilin (Predicted) | D3ZTC5 | 4 | 0.866681519 | 0.174066 | 0.758543339 | 0.00943505 |
|  | Protein-glutamine gamma-glutamyltransferase K | D3ZTH9 | 2 | 0.710079617 | 0.00115067 | 0.589069901 | 6.82E-07 |
|  | Protein Col6a1 | D3ZUL3 | 21 | 0.812216771 | 0.0482358 | 0.937953861 | 0.544878 |
|  | L-2-hydroxyglutarate dehydrogenase (Predicted) | D3ZVS2 | 10 | 1.231104124 | 0.0134636 | 1.395920203 | 0.000207692 |
|  | Protein Magmas | D3ZZV1 | 2 | 0.762137675 | 0.00989958 | 0.851430336 | 0.13032 |
|  | Protein Tecrl | D4A1Y9 | 4 | 1.051604243 | 0.550545 | 1.248157457 | 0.0137692 |
|  | Mitochondrial ribosomal protein L15 (Predicted), isoform CRA_b | D4A4B1 | 2 | 0.769331249 | 0.0127689 | 0.834271309 | 0.0884342 |
|  | Protein Opa3 | D4A4M6 | 3 | 1.259803631 | 0.00604485 | 1.560361457 | 7.43E-07 |
|  | Uncharacterized protein | D4A6I2 | 6 | 1.667722677 | 1.19E-09 | 0.948599074 | 0.617324 |
|  | Monoglyceride lipase | D4A892 | 7 | 0.794687414 | 0.0290821 | 0.929685201 | 0.491157 |
|  | Saccharopine dehydrogenase-like oxidoreductase | D4A8I1 | 2 | 1.197619278 | 0.0320893 | 1.222407803 | 0.0256787 |
|  | Protein Pm20d2 | D4AAB5 | 3 | 0.788692769 | 0.024181 | 0.892748654 | 0.285268 |
|  | Integrin alpha 5 (Mapped) | D4ACU9 | 2 | 0.829001172 | 0.0748887 | 0.802435889 | 0.0385944 |
|  | Striatin-3 | E9PT82 | 2 | 0.81203221 | 0.0479917 | 0.852526274 | 0.133412 |
|  | Protein Tnnc1 | E9PTA1 | 12 | 1.207594612 | 0.0249646 | 1.175879294 | 0.0720518 |
|  | Alpha-2-HS-glycoprotein | F1LM19 | 6 | 0.667984302 | 0.000127688 | 0.654639128 | 6.98E-05 |
|  | Myosin-4 | F1LMU0 | 3 | 1.243830432 | 0.0094983 | 1.377236387 | 0.000371732 |
|  | Fetuin-B | F1LN83 | 6 | 0.60668861 | 2.09E-06 | 0.645273061 | 3.92E-05 |
|  | Hydroxyacylglutathione hydrolase, mitochondrial | F1LQI1 | 5 | 1.127472434 | 0.154028 | 1.21919437 | 0.0276852 |
|  | GMP reductase | F1LRV6 | 6 | 1.228961531 | 0.0142639 | 1.302937767 | 0.00326452 |
|  | Integrin alpha-1 (Fragment) | F1LSR5 | 2 | 0.806828914 | 0.0415043 | 0.866081687 | 0.176094 |
|  | Protein LOC681849 (Fragment) | F1LWR6 | 2 | 0.797511144 | 0.0316577 | 0.823591562 | 0.0680751 |
|  | Protein Col4a2 (Fragment) | F1M6Q3 | 3 | 0.734605292 | 0.00340555 | 0.904440898 | 0.343859 |
|  | Uncharacterized protein (Fragment) | F1M7F8 | 2 | 1.204781699 | 0.0268141 | 1.013152793 | 0.888887 |
|  | Protein Cfh | F1M983 | 6 | 0.763696784 | 0.0104696 | 0.633164681 | 1.79E-05 |
|  | Protein RGD1310507 | F1MAG6 | 7 | 0.800065908 | 0.0341454 | 0.744229179 | 0.0055289 |
|  | Tryptophan--tRNA ligase, cytoplasmic | F8WFH8 | 3 | 1.606296208 | 1.75E-08 | 1.003918759 | 0.969904 |
|  | Histidine-rich glycoprotein | F8WFX4 | 5 | 0.637206978 | 1.88E-05 | 0.674423363 | 0.000217402 |
|  | Protein LOC100363782 | G3V6H0 | 2 | 1.243506622 | 0.00958418 | 1.250885551 | 0.0128627 |
|  | 4-trimethylaminobutyraldehyde dehydrogenase | G3V6J0 | 6 | 1.192107644 | 0.0367586 | 1.210680171 | 0.0336987 |
|  | Calumenin | G3V6S3 | 3 | 0.952119905 | 0.640587 | 0.726364684 | 0.00267965 |
|  | 2,4-dienoyl CoA reductase 1, mitochondrial, isoform CRA_a | G3V734 | 6 | 1.226401793 | 0.0152768 | 1.29436256 | 0.00412925 |
|  | Pyruvate dehydrogenase kinase, isoenzyme 4 | G3V778 | 6 | 0.66067673 | 8.31E-05 | 1.364451647 | 0.000549275 |
|  | Ceruloplasmin | G3V7K3 | 14 | 0.901486896 | 0.324359 | 0.629565299 | 1.40E-05 |
|  | Coagulation factor XIII A chain | G3V811 | 2 | 1.420633645 | 2.99E-05 | 0.947765938 | 0.611535 |
|  | Coagulation factor II, isoform CRA_a | G3V843 | 7 | 0.710144592 | 0.00115419 | 0.704134823 | 0.000988807 |
|  | Myosin-6 | G3V885 | 44 | 0.998173623 | 0.985208 | 1.304038857 | 0.0031667 |
|  | Glycerol-3-phosphate acyltransferase 1, mitochondrial | G3V9J8 | 2 | 0.806114635 | 0.0406716 | 1.138028104 | 0.151473 |
|  | 6-phosphofructokinase | G8JLS1 | 2 | 0.939968115 | 0.555999 | 0.777717136 | 0.0181847 |
|  | AMP deaminase 3 | AMPD3 | 2 | 1.221868998 | 0.0172324 | 1.180112123 | 0.0659425 |
|  | Glycerol-3-phosphate dehydrogenase [NAD(+)], cytoplasmic | GPDA | 6 | 1.136917943 | 0.127341 | 1.303647445 | 0.00320116 |
|  | Four and a half LIM domains protein 2 | FHL2 | 8 | 0.902144584 | 0.327769 | 0.690658768 | 0.000511113 |
|  | Peroxiredoxin-6 | PRDX6 | 12 | 1.190629978 | 0.0381086 | 1.200886217 | 0.0420337 |
|  | Ectonucleoside triphosphate diphosphohydrolase 2 | ENTP2 | 2 | 0.428442502 | 8.55E-16 | 0.575183823 | 2.10E-07 |
|  | Angiotensinogen | ANGT | 4 | 0.793881222 | 0.0283791 | 0.79461669 | 0.0307502 |
|  | Complement C3 | CO3 | 37 | 0.811373706 | 0.0471287 | 0.555253915 | 3.37E-08 |
|  | Ig kappa chain C region, B allele | KACB | 2 | 0.888917679 | 0.263223 | 0.619490997 | 6.98E-06 |
|  | Ig kappa chain C region, A allele | KACA | 2 | 0.411164381 | 3.26E-17 | 0.474588663 | 2.70E-12 |
|  | Hemoglobin subunit alpha-1/2 | HBA | 10 | 0.22516745 | 1.82E-45 | 0.428665887 | 1.92E-15 |
|  | Apolipoprotein E | APOE | 3 | 0.594693323 | 8.06E-07 | 0.666733522 | 0.000141575 |
|  | Isoform Gamma-A of Fibrinogen gamma chain | FIBG | 9 | 0.610414608 | 2.78E-06 | 0.486453519 | 1.37E-11 |
|  | Alpha-1-acid glycoprotein | A1AG | 3 | 3.317264281 | 3.87E-46 | 0.684294078 | 0.000368514 |
|  | Transthyretin | TTHY | 2 | 0.735628662 | 0.00355314 | 0.793546092 | 0.0297855 |
|  | Serum albumin | ALBU | 41 | 0.663126996 | 9.61E-05 | 0.749133162 | 0.00666951 |
|  | Ferritin light chain 1 | FRIL1 | 7 | 0.838829471 | 0.0950617 | 1.208942124 | 0.0350606 |
|  | Vitamin D-binding protein | VTDB | 5 | 0.941927794 | 0.569337 | 0.73428011 | 0.00372371 |
|  | Apolipoprotein A-I | APOA1 | 8 | 0.44704446 | 2.13E-14 | 0.461989199 | 4.33E-13 |
|  | Glutathione S-transferase alpha-3 | GSTA3 | 3 | 0.769164837 | 0.0126953 | 0.913596586 | 0.394138 |
|  | Isoform 4 of Fibronectin | FINC | 6 | 0.787202244 | 0.0230747 | 0.684780272 | 0.000377974 |
|  | Serine protease inhibitor A3L | SPA3L | 6 | 0.819759634 | 0.0590867 | 0.651043405 | 5.61E-05 |
|  | Serine protease inhibitor A3K | SPA3K | 3 | 0.758745351 | 0.00875031 | 0.687171701 | 0.000427789 |
|  | Haptoglobin | HPT | 12 | 1.325702561 | 0.000803056 | 0.673180272 | 0.000203057 |
|  | Beta-2-microglobulin | B2MG | 2 | 1.509356111 | 9.84E-07 | 1.005098409 | 0.959477 |
|  | Sodium/potassium-transporting ATPase subunit beta-1 | AT1B1 | 4 | 1.089365164 | 0.309383 | 1.360363541 | 0.000621449 |
|  | Peroxisomal acyl-coenzyme A oxidase 1 | ACOX1 | 7 | 0.816832679 | 0.054669 | 1.258923158 | 0.0105007 |
|  | Aldose reductase | ALDR | 9 | 1.293654723 | 0.00220824 | 1.229192004 | 0.0218662 |
|  | Complement C4 | CO4 | 14 | 0.780763823 | 0.0187662 | 0.653371237 | 6.46E-05 |
|  | Galectin-3 | LEG3 | 3 | 0.794640939 | 0.0290412 | 0.789817664 | 0.0266173 |
|  | T-kininogen 2 | KNT2 | 4 | 1.703627257 | 2.38E-10 | 0.608177648 | 3.05E-06 |
|  | Isoform LMW of Kininogen-1 | KNG1 | 2 | 0.570271904 | 9.72E-08 | 0.588228824 | 6.36E-07 |
|  | Serine protease inhibitor A3N | SPA3N | 8 | 1.393899142 | 7.87E-05 | 0.755536397 | 0.0084606 |
|  | ATP synthase protein 8 | ATP8 | 2 | 1.052434146 | 0.544299 | 1.206121103 | 0.0373758 |
|  | NADH-ubiquinone oxidoreductase chain 2 | NU2M | 2 | 1.047785006 | 0.579805 | 1.873457113 | 2.81E-12 |
|  | Pyruvate kinase isozymes M1/M2 | KPYM | 2 | 1.222346618 | 0.0170162 | 1.153762496 | 0.112446 |
|  | Serotransferrin | TRFE | 24 | 0.966625232 | 0.746416 | 0.706146302 | 0.00108714 |
|  | Alpha-1-inhibitor 3 | A1I3 | 4 | 0.651935724 | 4.87E-05 | 0.730526484 | 0.00319098 |
|  | Fibrinogen beta chain | FIBB | 11 | 0.548411941 | 1.18E-08 | 0.462018886 | 4.35E-13 |
|  | Isoform PAM-3B of Peptidyl-glycine alpha-amidating monooxygenase | AMD | 2 | 0.825391886 | 0.0683702 | 0.801666868 | 0.0377578 |
|  | Beta-enolase | ENOB | 8 | 1.427369785 | 2.33E-05 | 1.358498035 | 0.000657314 |
|  | Phosphoglycerate mutase 2 | PGAM2 | 9 | 1.223633252 | 0.0164457 | 1.284117859 | 0.00544251 |
|  | Alpha-1-antiproteinase | A1AT | 11 | 0.953932914 | 0.65356 | 0.649934937 | 5.24E-05 |
|  | Hemopexin | HEMO | 13 | 1.27825871 | 0.00351943 | 0.762633724 | 0.0109136 |
|  | Ig gamma-1 chain C region | IGHG1 | 5 | 0.680897542 | 0.000263117 | 0.711894732 | 0.00141813 |
|  | Ig gamma-2A chain C region | IGG2A | 7 | 0.527936964 | 1.33E-09 | 0.525039458 | 1.49E-09 |
|  | Ig gamma-2B chain C region | IGG2B | 4 | 0.674749798 | 0.000187545 | 0.644587491 | 3.75E-05 |
|  | Ig gamma-2C chain C region | IGG2C | 3 | 0.644858899 | 3.11E-05 | 0.849519149 | 0.125052 |
|  | Ig lambda-2 chain C region | LAC2 | 3 | 0.562534649 | 4.72E-08 | 0.470222792 | 1.45E-12 |
|  | Isoform Kidney of Band 3 anion transport protein | B3AT | 4 | 0.34857329 | 1.46E-23 | 0.468872594 | 1.19E-12 |
|  | Heme oxygenase 2 | HMOX2 | 2 | 0.681373547 | 0.000269992 | 0.760760799 | 0.0102138 |
|  | Glutathione peroxidase 3 | GPX3 | 3 | 1.368747428 | 0.000190138 | 1.132792481 | 0.16667 |
|  | Cathepsin D | CATD | 6 | 0.778968962 | 0.0176927 | 0.976077667 | 0.8166 |
|  | Carbonic anhydrase 2 | CAH2 | 4 | 0.389401182 | 3.46E-19 | 0.545466461 | 1.29E-08 |
|  | Proteasome subunit beta type-8 | PSB8 | 2 | 1.336025301 | 0.000573199 | 1.064222471 | 0.491781 |
|  | Proteasome subunit beta type-9 | PSB9 | 2 | 1.581411613 | 5.05E-08 | 0.820938683 | 0.0636309 |
|  | Peptidyl-prolyl cis-trans isomerase F, mitochondrial | PPIF | 5 | 0.948899511 | 0.617754 | 1.221339989 | 0.0263304 |
|  | Transgelin | TAGL | 6 | 0.785044002 | 0.0215474 | 0.79879828 | 0.0347652 |
|  | Isoform Short of 14-3-3 protein beta/alpha | 1433B | 3 | 0.882258195 | 0.233975 | 0.805742684 | 0.0423638 |
|  | Acylphosphatase-2 | ACYP2 | 2 | 1.297516523 | 0.00196054 | 1.17108911 | 0.0795466 |
|  | Signal transducer and activator of transcription 3 | STAT3 | 6 | 1.224358106 | 0.0161321 | 1.014417953 | 0.877914 |
|  | Alpha-soluble NSF attachment protein | SNAA | 2 | 1.220559627 | 0.0178383 | 1.180603483 | 0.0652631 |
|  | Serum paraoxonase/arylesterase 1 | PON1 | 2 | 0.324162786 | 1.09E-26 | 0.371180865 | 1.45E-20 |
|  | Phytanoyl-CoA dioxygenase, peroxisomal | PAHX | 6 | 0.739759632 | 0.00420739 | 1.085709095 | 0.362756 |
|  | Isoform 2 of Tropomyosin beta chain | TPM2 | 3 | 0.802856813 | 0.0370422 | 0.725350048 | 0.00256653 |
|  | 40S ribosomal protein S6 | RS6 | 4 | 0.745344473 | 0.00525792 | 0.883846575 | 0.245025 |
|  | Dynein light chain 1, cytoplasmic | DYL1 | 2 | 0.807328382 | 0.0420948 | 0.831038387 | 0.0818383 |
|  | cAMP-dependent protein kinase inhibitor alpha | IPKA | 2 | 0.736409054 | 0.00366941 | 0.392863978 | 1.88E-18 |
|  | Actin, alpha skeletal muscle | ACTS | 2 | 0.957330051 | 0.678082 | 1.377589975 | 0.000367705 |
|  | Acyl-protein thioesterase 1 | LYPA1 | 2 | 1.214092019 | 0.0211235 | 1.140207798 | 0.145487 |
|  | Short/branched chain specific acyl-CoA dehydrogenase, mitochondrial | ACDSB | 2 | 0.784011902 | 0.0208472 | 0.895877152 | 0.300312 |
|  | Mature alpha chain of major histocompatibility complex class I antigen (Fragment) | P79599 | 3 | 1.724366069 | 9.25E-11 | 0.914383966 | 0.398636 |
|  | Isoform 3 of Mitochondrial fission 1 protein | FIS1 | 2 | 1.415909966 | 3.55E-05 | 1.4281211 | 7.39E-05 |
|  | Kallistatin | P97569 | 4 | 0.704840621 | 0.000896564 | 0.762118299 | 0.010717 |
|  | Plasminogen | PLMN | 4 | 0.609400967 | 2.58E-06 | 0.687018628 | 0.00042443 |
|  | NADH-ubiquinone oxidoreductase chain 2 | Q06QE9 | 2 | 1.509308686 | 9.86E-07 | 1.312535713 | 0.00249939 |
|  | Vitronectin | Q3KR94 | 4 | 0.83119444 | 0.0790769 | 0.649474558 | 5.09E-05 |
|  | Desmocollin 2 | Q3T1K6 | 3 | 0.789348524 | 0.0246814 | 0.871839421 | 0.196788 |
|  | Peripherin | Q496Z5 | 2 | 0.78483688 | 0.0214053 | 1.149509865 | 0.122065 |
|  | DNAation factor, alpha subunit | Q498U6 | 2 | 1.24556204 | 0.00905095 | 1.090627148 | 0.33682 |
|  | Protein Tpd52l1 | Q499Q2 | 2 | 0.579465895 | 2.22E-07 | 0.662571899 | 0.000111498 |
|  | Proteasome subunit beta type-10 | PSB10 | 2 | 1.824472769 | 8.69E-13 | 0.877545602 | 0.218849 |
|  | Protein FAM162A | F162A | 6 | 1.017841318 | 0.83458 | 1.201258531 | 0.0416863 |
|  | Ribosomal protein L11 | Q4V8I6 | 2 | 1.213168331 | 0.0216349 | 1.137082824 | 0.154131 |
|  | UDP-glucose pyrophosphorylase 2 | Q4V8I9 | 11 | 1.207041726 | 0.0253188 | 1.00025295 | 0.997973 |
|  | Golgi phosphoprotein 3 | Q569C9 | 3 | 1.023517021 | 0.783342 | 0.768040146 | 0.0131669 |
|  | Kynurenine--oxoglutarate transaminase 3 | KAT3 | 8 | 1.153192829 | 0.0903193 | 1.210397259 | 0.0339171 |
|  | Nicotinamide nucleotide transhydrogenase | Q5BJZ3 | 27 | 0.78668835 | 0.0227032 | 0.993902286 | 0.95036 |
|  | Glutathione S-transferase mu 4 | Q5BK56 | 2 | 1.27659649 | 0.00369812 | 0.950699248 | 0.632001 |
|  | Col4a1 protein (Fragment) | Q5FWY9 | 4 | 0.752098692 | 0.00682695 | 0.917406665 | 0.41615 |
|  | GTP-binding protein SAR1b | SAR1B | 2 | 1.057909833 | 0.504105 | 1.285026815 | 0.00531187 |
|  | Apolipoprotein H | Q5I0M1 | 7 | 0.718469797 | 0.00169327 | 0.756347872 | 0.00871466 |
|  | Pirin | PIR | 2 | 1.143344275 | 0.11145 | 1.225307499 | 0.0239814 |
|  | Kininogen 1 | Q5PQU1 | 4 | 0.9649149 | 0.733722 | 0.583241253 | 4.20E-07 |
|  | Igh-6 protein (Fragment) | Q5RK07 | 4 | 0.504200808 | 7.99E-11 | 0.506546693 | 1.75E-10 |
|  | Eukaryotic translation initiation factor 4B | Q5RKG9 | 3 | 0.902509156 | 0.329668 | 0.698369473 | 0.000749575 |
|  | Enabled homolog (Drosophila) | Q5XHX3 | 5 | 0.912642378 | 0.384952 | 0.795297084 | 0.0313764 |
|  | Alanyl-tRNA editing protein Aarsd1 | AASD1 | 3 | 0.842095355 | 0.102595 | 0.810989397 | 0.0489443 |
|  | Complement component C9 | CO9 | 6 | 1.301924182 | 0.0017099 | 0.85287992 | 0.134421 |
|  | Alpha-1-macroglobulin | A1M | 23 | 0.487855219 | 9.52E-12 | 0.457504703 | 2.20E-13 |
|  | Ribosomal protein L19 (Fragment) | Q63287 | 2 | 0.73233077 | 0.00309667 | 0.88946983 | 0.270002 |
|  | Inter-alpha-trypsin inhibitor heavy chain H3 | ITIH3 | 8 | 0.741961827 | 0.00459743 | 0.69940035 | 0.00078809 |
|  | Proteasome activator complex subunit 1 | PSME1 | 12 | 1.429011494 | 2.19E-05 | 1.023120749 | 0.803543 |
|  | Alpha globin | Q63910 | 4 | 0.53357215 | 2.47E-09 | 0.71532501 | 0.00165595 |
|  | Heparin cofactor 2 | HEP2 | 4 | 0.758820262 | 0.00877439 | 0.773628293 | 0.0158977 |
|  | Ubiquinone biosynthesis protein COQ9, mitochondrial | COQ9 | 7 | 1.069800307 | 0.423146 | 1.264671846 | 0.0090638 |
|  | Phosphoribosyl pyrophosphate synthase-associated protein 2 | Q6AZ40 | 2 | 1.010712384 | 0.900336 | 1.217306024 | 0.028929 |
|  | Carboxypeptidase Q | CBPQ | 2 | 0.894761866 | 0.29069 | 0.745824232 | 0.00587974 |
|  | Rat glutathione S-transferase | Q6LDP3 | 4 | 0.79607318 | 0.0303238 | 0.80179496 | 0.0378961 |
|  | B-factor, properdin | Q6MG74 | 8 | 0.864252146 | 0.165779 | 0.703538082 | 0.000961209 |
|  | Keratin, type II cytoskeletal 5 | K2C5 | 3 | 1.269181909 | 0.00460345 | 0.7795801 | 0.0193146 |
|  | Glutathione peroxidase | Q6PDW8 | 3 | 1.83192949 | 6.09E-13 | 0.971762853 | 0.784502 |
|  | Erythroid spectrin beta | Q6XDA0 | 9 | 0.708283994 | 0.00105712 | 0.73481955 | 0.00380635 |
|  | Mitochondrial import receptor subunit TOM70 | TOM70 | 3 | 1.014913158 | 0.86142 | 1.290451664 | 0.00459104 |
|  | Bone marrow stromal antigen 2 | BST2 | 2 | 1.144454394 | 0.10888 | 0.792987089 | 0.0292917 |
|  | Isoform Cytoplasmic+peroxisomal of Malonyl-CoA decarboxylase, mitochondrial | DCMC | 6 | 1.123564088 | 0.166313 | 1.291911779 | 0.00441326 |
|  | Small muscular protein | SMPX | 2 | 1.285346589 | 0.00284444 | 1.219326771 | 0.0275999 |
|  | Signal transducer and activator of transcription 1 | Q9QXK0 | 17 | 2.805591078 | 1.35E-34 | 1.009412254 | 0.921524 |
|  | Myoglobin | MYG | 19 | 1.206884486 | 0.0254204 | 1.224330794 | 0.0245414 |
|  | CD151 antigen | CD151 | 2 | 1.142092621 | 0.114409 | 1.277328186 | 0.00651726 |
|  | Mitochondrial import inner membrane translocase subunit Tim8 A | TIM8A | 3 | 1.253695107 | 0.00719826 | 1.193317212 | 0.0496742 |
|  | Hydroxyacyl-coenzyme A dehydrogenase, mitochondrial | HCDH | 14 | 1.076405233 | 0.382021 | 1.314148559 | 0.00238871 |

**Table S-2A.** Enrichment of pathway maps in SP group.

| No. | Maps | Total*^a^* | p-Value*^b^* | Min FDR | p-value | FDR | In Data*^c^* | Network Objects from Active Data |
| --- | --- | --- | --- | --- | --- | --- | --- | --- |
| 1 | Blood coagulation_Blood coagulation | 39 | 1.88809E-08 | 3.66E-06 | 1.89E-08 | 3.66E-06 | 7 | Fibrinogen gamma, Bradykinin, Fibrinogen alpha, Fibrinogen (fibrin), Thrombin, Fibrinogen beta, KNG |
| 2 | Immune response_Lectin induced complement pathway | 50 | 2.47774E-06 | 0.000227 | 2.48E-06 | 0.000227 | 6 | C4, C4a, C9, Factor I, C4b, C8alpha |
| 3 | Immune response_Classical complement pathway | 53 | 3.51288E-06 | 0.000227 | 3.51E-06 | 0.000227 | 6 | C4, C4a, C9, Factor I, C4b, C8alpha |
| 4 | Protein folding and maturation_Bradykinin / Kallidin maturation | 32 | 4.72111E-06 | 0.000229 | 4.72E-06 | 0.000229 | 5 | des-Arg9-bradykinin, des-Arg10-kallidin, Bradykinin, Kallidin, KNG |
| 5 | Transport_HDL-mediated reverse cholesterol transport | 44 | 2.36401E-05 | 0.000917 | 2.36E-05 | 0.000917 | 5 | Pre beta-1 HDL, APOE, APOA1, Nascent HDL, Large apoE-rich HDL |
| 6 | Cell adhesion_Cell-matrix glycoconjugates | 38 | 0.000221526 | 0.007163 | 0.000222 | 0.007163 | 4 | A1M, ITIH, ITIH3, ECM1 |
| 7 | Niacin-HDL metabolism | 46 | 0.000466786 | 0.012937 | 0.000467 | 0.012937 | 4 | APOB, LDL, HDL proteins, APOA1 |
| 8 | Role of ZNF202 in regulation of expression of genes involved in atherosclerosis | 21 | 0.000579022 | 0.014041 | 0.000579 | 0.014041 | 3 | HDL proteins, APOE, APOA4 |
| 9 | Immune response_Antigen presentation by MHC class I | 28 | 0.001370408 | 0.02954 | 0.00137 | 0.02954 | 3 | Beta-2-microglobulin, Tapasin, PSME1 |
| 10 | Transport_Low density lipoproteins assembly and remodeling | 34 | 0.002419177 | 0.046401 | 0.002419 | 0.046401 | 3 | APOB, Large LDL, Small LDL |
| 11 | Cell adhesion_Plasmin signaling | 35 | 0.002630975 | 0.046401 | 0.002631 | 0.046401 | 3 | Fibrinogen (fibrin), PZP, Collagen IV |
| 12 | Immune response_Alternative complement pathway | 53 | 0.008503586 | 0.131144 | 0.008504 | 0.131144 | 3 | C9, Factor I, C8alpha |
| 13 | Immune response_Oncostatin M signaling via JAK-Stat in mouse cells | 18 | 0.008787999 | 0.131144 | 0.008788 | 0.131144 | 2 | STAT1, SERPINA3 (ACT) |
| 14 | Immune response_Oncostatin M signaling via JAK-Stat in human cells | 20 | 0.010801962 | 0.149684 | 0.010802 | 0.149684 | 2 | STAT1, SERPINA3 (ACT) |
| 15 | Transcription_Role of VDR in regulation of genes involved in osteoporosis | 61 | 0.01248765 | 0.149738 | 0.012488 | 0.149738 | 3 | COL1A1, Carbonic anhydrase II, APOE |
| 16 | Development_Thrombopoetin signaling via JAK-STAT pathway | 22 | 0.012999204 | 0.149738 | 0.012999 | 0.149738 | 2 | STAT1, SERPINA3 (ACT) |
| 17 | Glycolysis and gluconeogenesis p.3 / Human version | 24 | 0.015373575 | 0.149738 | 0.015374 | 0.149738 | 2 | ENO, ENO3 |
| 18 | Cholesterol and Sphingolipid transport / Recycling to plasma membrane in lung (normal and CF) | 24 | 0.015373575 | 0.149738 | 0.015374 | 0.149738 | 2 | HDL proteins, APOA1 |
| 19 | Glycolysis and gluconeogenesis p.3 | 24 | 0.015373575 | 0.149738 | 0.015374 | 0.149738 | 2 | ENO, ENO3 |
| 20 | Role of Tissue factor-induced Thrombin signaling in cancerogenesis | 66 | 0.015436856 | 0.149738 | 0.015437 | 0.149738 | 3 | Fibrinogen alpha, Fibrinogen (fibrin), Thrombin |
| 21 | Immune response_LPS-induced platelet activation | 25 | 0.016625301 | 0.153586 | 0.016625 | 0.153586 | 2 | Fibrinogen (fibrin), Thrombin |
| 22 | Development_Growth hormone signaling via STATs and PLC/IP3 | 35 | 0.031337411 | 0.237204 | 0.031337 | 0.237204 | 2 | STAT1, Fibrinogen beta |
| 23 | Immune response_Role of the Membrane attack complex in cell survival | 35 | 0.031337411 | 0.237204 | 0.031337 | 0.237204 | 2 | C9, C8alpha |
| 24 | Nitrogen metabolism | 35 | 0.031337411 | 0.237204 | 0.031337 | 0.237204 | 2 | Carbonic anhydrase II, Carbonic anhydrase I |
| 25 | Androstenedione and testosterone biosynthesis and metabolism p.2 | 35 | 0.031337411 | 0.237204 | 0.031337 | 0.237204 | 2 | AKR1C1, AKR1C2 |
| 26 | Androstenedione and testosterone biosynthesis and metabolism p.2/ Rodent version | 36 | 0.033012912 | 0.237204 | 0.033013 | 0.237204 | 2 | AKR1C1, AKR1C2 |
| 27 | Nitrogen metabolism/ Rodent version | 36 | 0.033012912 | 0.237204 | 0.033013 | 0.237204 | 2 | Carbonic anhydrase II, Carbonic anhydrase I |
| 28 | Regulation of metabolism_Bile acids regulation of glucose and lipid metabolism via FXR | 37 | 0.034722901 | 0.24058 | 0.034723 | 0.24058 | 2 | APOB, APOE |
| 29 | Transcription_Role of AP-1 in regulation of cellular metabolism | 38 | 0.036466739 | 0.24395 | 0.036467 | 0.24395 | 2 | HBB, Alpha1-globin |
| 30 | Development_EPO-induced PI3K/AKT pathway and Ca(2+) influx | 43 | 0.045671868 | 0.276886 | 0.045672 | 0.276886 | 2 | HBB, Alpha1-globin |
| 31 | Role of platelets in allograft rejection | 43 | 0.045671868 | 0.276886 | 0.045672 | 0.276886 | 2 | Fibrinogen (fibrin), Thrombin |
| 32 | Role of platelets in the initiation of in-stent restenosis | 43 | 0.045671868 | 0.276886 | 0.045672 | 0.276886 | 2 | Fibrinogen (fibrin), Thrombin |
| 33 | Heme metabolism | 103 | 0.048515276 | 0.285211 | 0.048515 | 0.285211 | 3 | Holotransferrin, Heme oxygenase 2, Apotransferrin |

*^a^* The total number of proteins in the pathway.

*^b^* Probability of identified differential protein list associates to the generated pathway.

*^c^* The number of proteins derived from proteomics analysis.

**Table S -2B.** Enrichment of pathway maps in SPD group.

| No. | Maps | Total*^a^* | p-Value*^b^* | Min FDR | p-value | FDR | In Data*^c^* | Network Objects from Active Data |
| --- | --- | --- | --- | --- | --- | --- | --- | --- |
| 1 | Immune response_Alternative complement pathway | 53 | 2.3E-24 | 3.66E-22 | 2.3E-24 | 3.66E-22 | 18 | Factor H, C3b, C3dg, C5, Vitronectin, C3a, Factor B, C5a, C3, C5 convertase (C3b2Bb), Factor I, Factor Ba, C3c, C5b, Factor Bb, C3 convertase (C3bBb), C8alpha, iC3b |
| 2 | Immune response_Lectin induced complement pathway | 50 | 1.78E-19 | 1.42E-17 | 1.78E-19 | 1.42E-17 | 15 | C3b, C3dg, C5, C4, C3a, C5a, C3, C4a, Factor I, C3c, C5b, C4b, C8alpha, iC3b, C1 inhibitor |
| 3 | Immune response_Classical complement pathway | 53 | 4.84E-19 | 2.57E-17 | 4.84E-19 | 2.57E-17 | 15 | C3b, C3dg, C5, C4, C3a, C5a, C3, C4a, Factor I, C3c, C5b, C4b, C8alpha, iC3b, C1 inhibitor |
| 4 | Blood coagulation_Blood coagulation | 39 | 3.36E-14 | 1.34E-12 | 3.36E-14 | 1.34E-12 | 11 | Fibrinogen gamma, Tissue factor, Bradykinin, Alpha 1-antitrypsin, SERPINF2, HC II, Fibrinogen alpha, Fibrinogen (fibrin), Thrombin, Fibrinogen beta, KNG |
| 5 | Complement pathway disruption in thrombotic microangiopathy | 37 | 3.59E-11 | 1.14E-09 | 3.59E-11 | 1.14E-09 | 9 | Factor H, C3b, C5, C3a, C5a, C3, C5 convertase (C3b2Bb), Factor I, C5b |
| 6 | Protein folding and maturation_Bradykinin / Kallidin maturation | 32 | 1.05E-05 | 0.000278 | 1.05E-05 | 0.000278 | 5 | des-Arg9-bradykinin, des-Arg10-kallidin, Bradykinin, Kallidin, KNG |
| 7 | Transport_HDL-mediated reverse cholesterol transport | 44 | 5.18E-05 | 0.001177 | 5.18E-05 | 0.001177 | 5 | Pre beta-1 HDL, APOE, APOA1, Nascent HDL, Large apoE-rich HDL |
| 8 | Cell adhesion_Plasmin signaling | 35 | 0.000299 | 0.005942 | 0.000299 | 0.005942 | 4 | Fibronectin, Fibrinogen (fibrin), PZP, C1 inhibitor |
| 9 | Niacin-HDL metabolism | 46 | 0.000862 | 0.014772 | 0.000862 | 0.014772 | 4 | APOB, LDL, HDL proteins, APOA1 |
| 10 | Role of ZNF202 in regulation of expression of genes involved in atherosclerosis | 21 | 0.000929 | 0.014772 | 0.000929 | 0.014772 | 3 | HDL proteins, APOE, APOA4 |
| 11 | Role of Tissue factor-induced Thrombin signaling in cancerogenesis | 66 | 0.003303 | 0.046842 | 0.003303 | 0.046842 | 4 | Tissue factor, Fibrinogen alpha, Fibrinogen (fibrin), Thrombin |
| 12 | Cytoskeleton remodeling_TGF, WNT and cytoskeletal remodeling | 111 | 0.003749 | 0.046842 | 0.003749 | 0.046842 | 5 | Fibronectin, Vitronectin, Destrin, Actin, C1 inhibitor |
| 13 | Transport_Low density lipoproteins assembly and remodeling | 34 | 0.00383 | 0.046842 | 0.00383 | 0.046842 | 3 | APOB, Large LDL, Small LDL |
| 14 | Blood coagulation_Platelet microparticle generation | 71 | 0.004299 | 0.048825 | 0.004299 | 0.048825 | 4 | Fibronectin, Tissue factor, Fibrinogen (fibrin), Thrombin |
| 15 | Cell adhesion_Cell-matrix glycoconjugates | 38 | 0.005256 | 0.055716 | 0.005256 | 0.055716 | 3 | A1M, ITIH, ITIH3 |
| 16 | Role of cell adhesion in vaso-occlusion in Sickle cell disease | 43 | 0.007436 | 0.065686 | 0.007436 | 0.065686 | 3 | Fibronectin, Fibrinogen (fibrin), iC3b |
| 17 | Role of platelets in allograft rejection | 43 | 0.007436 | 0.065686 | 0.007436 | 0.065686 | 3 | Tissue factor, Fibrinogen (fibrin), Thrombin |
| 18 | Role of platelets in the initiation of in-stent restenosis | 43 | 0.007436 | 0.065686 | 0.007436 | 0.065686 | 3 | Tissue factor, Fibrinogen (fibrin), Thrombin |
| 19 | High shear stress-induced platelet activation | 46 | 0.008965 | 0.075024 | 0.008965 | 0.075024 | 3 | Fibronectin, Fibrinogen (fibrin), Thrombin |
| 20 | Cytoskeleton remodeling_Cytoskeleton remodeling | 102 | 0.015123 | 0.112922 | 0.015123 | 0.112922 | 4 | Fibronectin, Vitronectin, Destrin, C1 inhibitor |
| 21 | Cell adhesion_Integrin inside-out signaling | 56 | 0.015315 | 0.112922 | 0.015315 | 0.112922 | 3 | Fibronectin, Fibrinogen (fibrin), Thrombin |
| 22 | Heme metabolism | 103 | 0.015624 | 0.112922 | 0.015624 | 0.112922 | 4 | Holotransferrin, Ceruloplasmin, Heme oxygenase 2, Apotransferrin |
| 23 | Expression targets of Tissue factor signaling in cancer | 22 | 0.017675 | 0.122185 | 0.017675 | 0.122185 | 2 | Tissue factor, Thrombin |
| 24 | Glycolysis and gluconeogenesis p.3 / Human version | 24 | 0.020865 | 0.122871 | 0.020865 | 0.122871 | 2 | ENO, ENO3 |
| 25 | Cell adhesion_Endothelial cell contacts by non-junctional mechanisms | 24 | 0.020865 | 0.122871 | 0.020865 | 0.122871 | 2 | Fibronectin, Vitronectin |
| 26 | Cholesterol and Sphingolipid transport / Recycling to plasma membrane in lung (normal and CF) | 24 | 0.020865 | 0.122871 | 0.020865 | 0.122871 | 2 | HDL proteins, APOA1 |
| 27 | Glycolysis and gluconeogenesis p.3 | 24 | 0.020865 | 0.122871 | 0.020865 | 0.122871 | 2 | ENO, ENO3 |
| 28 | Immune response_LPS-induced platelet activation | 25 | 0.022543 | 0.128013 | 0.022543 | 0.128013 | 2 | Fibrinogen (fibrin), Thrombin |
| 29 | Development_S1P2 and S1P3 receptors in cell proliferation and differentiation | 26 | 0.024276 | 0.130397 | 0.024276 | 0.130397 | 2 | Transgelin, Actin |
| 30 | Development_Growth factors in regulation of oligodendrocyte precursor cell proliferation | 67 | 0.024603 | 0.130397 | 0.024603 | 0.130397 | 3 | Fibronectin, Neuregulin 1, Vitronectin |
| 31 | Platelet activation during ADAM-TS13-deficient thrombotic microangiopathy development | 28 | 0.027897 | 0.143086 | 0.027897 | 0.143086 | 2 | Fibronectin, Fibrinogen (fibrin) |
| 32 | Development_Transcription factors in segregation of hepatocytic lineage | 30 | 0.031721 | 0.15284 | 0.031721 | 0.15284 | 2 | Alpha 1-antitrypsin, Albumin |
| 33 | Development_Slit-Robo signaling | 30 | 0.031721 | 0.15284 | 0.031721 | 0.15284 | 2 | MENA, Actin |
| 34 | Cell adhesion_Alpha-4 integrins in cell migration and adhesion | 34 | 0.039943 | 0.185991 | 0.039943 | 0.185991 | 2 | Fibronectin, GIT1 |
| 35 | Immune response_Role of the Membrane attack complex in cell survival | 35 | 0.042111 | 0.185991 | 0.042111 | 0.185991 | 2 | C5, C8alpha |
| 36 | Nitrogen metabolism | 35 | 0.042111 | 0.185991 | 0.042111 | 0.185991 | 2 | Carbonic anhydrase II, Carbonic anhydrase I |
| 37 | Nitrogen metabolism/ Rodent version | 36 | 0.044323 | 0.189892 | 0.044323 | 0.189892 | 2 | Carbonic anhydrase II, Carbonic anhydrase I |
| 38 | Cell adhesion_Role of tetraspanins in the integrin-mediated cell adhesion | 37 | 0.046577 | 0.189892 | 0.046577 | 0.189892 | 2 | Fibronectin, Actin |
| 39 | Regulation of metabolism_Bile acids regulation of glucose and lipid metabolism via FXR | 37 | 0.046577 | 0.189892 | 0.046577 | 0.189892 | 2 | APOB, APOE |
| 40 | Transcription_Role of AP-1 in regulation of cellular metabolism | 38 | 0.048873 | 0.19427 | 0.048873 | 0.19427 | 2 | HBB, Alpha1-globin |

*^a^* The total number of proteins in the pathway.

*^b^* Probability of identified differential protein list associates to the generated pathway.

*^c^* The number of proteins derived from proteomics analysis.

**Table S -2C.** Enrichment of pathway maps both in SP and SPD groups.

| No. | Maps | Total*^a^* | SP p-Value*^b^* | SPD p-Value*^b^* | SP In Data*^c^* | SPD In Data*^c^* | SP Network Objects from Active Data | SPD Network Objects from Active Data |
| --- | --- | --- | --- | --- | --- | --- | --- | --- |
| 1 | Blood coagulation_Blood coagulation | 39 | 1.89E-08 | 3.36366E-14 | 7 | 11 | Fibrinogen gamma, Bradykinin, Fibrinogen alpha, Fibrinogen (fibrin), Thrombin, Fibrinogen beta, KNG | Fibrinogen gamma, Tissue factor, Bradykinin, Alpha 1-antitrypsin, SERPINF2, HC II, Fibrinogen alpha, Fibrinogen (fibrin), Thrombin, Fibrinogen beta, KNG |
| 2 | Immune response_Lectin induced complement pathway | 50 | 2.48E-06 | 1.78223E-19 | 6 | 15 | C4, C4a, C9, Factor I, C4b, C8alpha | C3b, C3dg, C5, C4, C3a, C5a, C3, C4a, Factor I, C3c, C5b, C4b, C8alpha, iC3b, C1 inhibitor |
| 3 | Immune response_Classical complement pathway | 53 | 3.51E-06 | 4.84174E-19 | 6 | 15 | C4, C4a, C9, Factor I, C4b, C8alpha | C3b, C3dg, C5, C4, C3a, C5a, C3, C4a, Factor I, C3c, C5b, C4b, C8alpha, iC3b, C1 inhibitor |
| 4 | Protein folding and maturation_Bradykinin / Kallidin maturation | 32 | 4.72E-06 | 1.0493E-05 | 5 | 5 | des-Arg9-bradykinin, des-Arg10-kallidin, Bradykinin, Kallidin, KNG | des-Arg9-bradykinin, des-Arg10-kallidin, Bradykinin, Kallidin, KNG |
| 5 | Transport_HDL-mediated reverse cholesterol transport | 44 | 2.36E-05 | 5.18153E-05 | 5 | 5 | Pre beta-1 HDL, APOE, APOA1, Nascent HDL, Large apoE-rich HDL | Pre beta-1 HDL, APOE, APOA1, Nascent HDL, Large apoE-rich HDL |
| 6 | Cell adhesion_Cell-matrix glycoconjugates | 38 | 0.000222 | 0.005256236 | 4 | 3 | A1M, ITIH, ITIH3, ECM1 | A1M, ITIH, ITIH3 |
| 7 | Niacin-HDL metabolism | 46 | 0.000467 | 0.00086172 | 4 | 4 | APOB, LDL, HDL proteins, APOA1 | APOB, LDL, HDL proteins, APOA1 |
| 8 | Role of ZNF202 in regulation of expression of genes involved in atherosclerosis | 21 | 0.000579 | 0.000929084 | 3 | 3 | HDL proteins, APOE, APOA4 | HDL proteins, APOE, APOA4 |
| 9 | Transport_Low density lipoproteins assembly and remodeling | 34 | 0.002419 | 0.003829884 | 3 | 3 | APOB, Large LDL, Small LDL | APOB, Large LDL, Small LDL |
| 10 | Cell adhesion_Plasmin signaling | 35 | 0.002631 | 0.000298973 | 3 | 4 | Fibrinogen (fibrin), PZP, Collagen IV | Fibronectin, Fibrinogen (fibrin), PZP, C1 inhibitor |
| 11 | Immune response_Alternative complement pathway | 53 | 0.008504 | 2.30111E-24 | 3 | 18 | C9, Factor I, C8alpha | Factor H, C3b, C3dg, C5, Vitronectin, C3a, Factor B, C5a, C3, C5 convertase (C3b2Bb), Factor I, Factor Ba, C3c, C5b, Factor Bb, C3 convertase (C3bBb), C8alpha, iC3b |
| 12 | Glycolysis and gluconeogenesis p.3 / Human version | 24 | 0.015374 | 0.020864909 | 2 | 2 | ENO, ENO3 | ENO, ENO3 |
| 13 | Cholesterol and Sphingolipid transport / Recycling to plasma membrane in lung (normal and CF) | 24 | 0.015374 | 0.020864909 | 2 | 2 | HDL proteins, APOA1 | HDL proteins, APOA1 |
| 14 | Glycolysis and gluconeogenesis p.3 | 24 | 0.015374 | 0.020864909 | 2 | 2 | ENO, ENO3 | ENO, ENO3 |
| 15 | Role of Tissue factor-induced Thrombin signaling in cancerogenesis | 66 | 0.015437 | 0.003303387 | 3 | 4 | Fibrinogen alpha, Fibrinogen (fibrin), Thrombin | Tissue factor, Fibrinogen alpha, Fibrinogen (fibrin), Thrombin |
| 16 | Immune response_LPS-induced platelet activation | 25 | 0.016625 | 0.022543255 | 2 | 2 | Fibrinogen (fibrin), Thrombin | Fibrinogen (fibrin), Thrombin |
| 17 | Immune response_Role of the Membrane attack complex in cell survival | 35 | 0.031337 | 0.042111111 | 2 | 2 | C9, C8alpha | C5, C8alpha |
| 18 | Nitrogen metabolism | 35 | 0.031337 | 0.042111111 | 2 | 2 | Carbonic anhydrase II, Carbonic anhydrase I | Carbonic anhydrase II, Carbonic anhydrase I |
| 19 | Nitrogen metabolism/ Rodent version | 36 | 0.033013 | 0.044323011 | 2 | 2 | Carbonic anhydrase II, Carbonic anhydrase I | Carbonic anhydrase II, Carbonic anhydrase I |
| 20 | Regulation of metabolism_Bile acids regulation of glucose and lipid metabolism via FXR | 37 | 0.034723 | 0.046577249 | 2 | 2 | APOB, APOE | APOB, APOE |
| 21 | Transcription_Role of AP-1 in regulation of cellular metabolism | 38 | 0.036467 | 0.048872861 | 2 | 2 | HBB, Alpha1-globin | HBB, Alpha1-globin |
| 22 | Role of platelets in allograft rejection | 43 | 0.045672 | 0.007436105 | 2 | 3 | Fibrinogen (fibrin), Thrombin | Tissue factor, Fibrinogen (fibrin), Thrombin |
| 23 | Role of platelets in the initiation of in-stent restenosis | 43 | 0.045672 | 0.007436105 | 2 | 3 | Fibrinogen (fibrin), Thrombin | Tissue factor, Fibrinogen (fibrin), Thrombin |
| 24 | Heme metabolism | 103 | 0.048515 | 0.015624415 | 3 | 4 | Holotransferrin, Heme oxygenase 2, Apotransferrin | Holotransferrin, Ceruloplasmin, Heme oxygenase 2, Apotransferrin |

*^a^* The total number of proteins in the pathway.

*^b^* Probability of identified differential protein list associates to the generated pathway.

*^c^* The number of proteins derived from proteomics analysis.

**Table S-3.** Diﬀerential metabolites of SP vs O group filtered by VIP>1 and P-value<0.05.

| No. | Var ID (Primary) | PEAK | VIP | P-VALUE | FOLD CHANGE |
| --- | --- | --- | --- | --- | --- |
|  | 570 | Diglycerol 2 | 2.20803 | 1.05E-07 | 2313725 |
|  | 516 | pentadecanoic acid | 2.20416 | 0.000787 | 3889870 |
|  | 132 | phosphate | 2.04346 | 7.47E-06 | 4.722322 |
|  | 498 | 1,5-Anhydroglucitol | 1.98477 | 0.000356 | 133.4331 |
|  | 574 | Methylmalonic acid | 1.88364 | 0.001782 | 6.442636 |
|  | 34 | Methylmalonic acid | 1.8797 | 0.002828 | 5.902372 |
|  | 660 | Zymosterol 2 | 1.87752 | 0.030631 | 1.627695 |
|  | 637 | terephthalic acid | 1.86705 | 6.86E-05 | 0.09893 |
|  | 459 | 1,5-Anhydroglucitol | 1.86285 | 0.033698 | 13062601 |
|  | 196 | N-methylaniline | 1.86023 | 0.001269 | 0.181713 |
|  | 27 | Analyte 31 | 1.84512 | 0.000755 | 4.43E-07 |
|  | 170 | hydroxylamine | 1.80191 | 0.009059 | 1.472116 |
|  | 148 | proline | 1.78215 | 0.004664 | 0.526496 |
|  | 628 | Gluconic lactone 2 | 1.73127 | 0.0129 | 1.744108 |
|  | 108 | Carnitine | 1.71567 | 0.010814 | 2.763916 |
|  | 508 | lactose 1 | 1.71369 | 0.009087 | 2.641062 |
|  | 561 | thymidine 5'-monophosphate 1 | 1.68172 | 0.028376 | 1.243353 |
|  | 631 | sucrose | 1.63788 | 0.012341 | 1000639 |
|  | 82 | urea | 1.63538 | 0.024048 | 0.24947 |
|  | 645 | Tagatose 1 | 1.59504 | 0.01848 | 3.007636 |
|  | 547 | galactose 2 | 1.59418 | 0.014242 | 3.924616 |
|  | 202 | 2,3-Dihydroxypyridine | 1.58654 | 0.021909 | 2.284606 |
|  | 273 | nicotinic acid | 1.57762 | 0.004089 | 0.264098 |
|  | 299 | Menthone 2 | 1.57479 | 0.018604 | 13411849 |
|  | 584 | 2-Monoolein | 1.56002 | 0.002847 | 0.178304 |
|  | 517 | pentadecanoic acid | 1.55865 | 0.001467 | 0.215277 |
|  | 153 | butyraldehyde 2 | 1.5122 | 0.014148 | 1.07E-06 |
|  | 475 | D-Altrose 1 | 1.49705 | 0.045001 | 6.952884 |
|  | 323 | 5,6-Dimethylbenzimidazole 1 | 1.48267 | 0.016616 | 0.069681 |
|  | 386 | 2-ketoadipate 4 | 1.48132 | 0.021662 | 0.351282 |
|  | 197 | Salicylaldehyde | 1.44623 | 0.019106 | 0.193347 |
|  | 136 | 2,6-Diaminopimelic acid 2 | 1.3937 | 0.00026 | 4.550109 |
|  | 243 | oxalic acid | 1.36341 | 0.033457 | 1.707395 |
|  | 305 | 21-hydroxypregnenolone 1 | 1.35911 | 0.012179 | 0.154897 |
|  | 449 | conduritol b epoxide 2 | 1.34757 | 0.049618 | 1299764 |
|  | 501 | 3,6-Anhydro-D-galactose 1 | 1.32995 | 0.022278 | 2.246214 |
|  | 373 | Methoxamedrine 1 | 1.31904 | 0.001497 | 0.283859 |
|  | 133 | phosphate | 1.29637 | 0.019396 | 0.392955 |
|  | 546 | tetracosane | 1.27774 | 0.026229 | 0.426074 |
|  | 212 | creatine degr | 1.27684 | 0.00889 | 0.348801 |
|  | 268 | asparagine 3 | 1.26631 | 0.036973 | 0.461247 |
|  | 548 | methyl heptadecanoate | 1.26385 | 0.018781 | 0.414384 |
|  | 50 | Methylmalonic acid | 1.24651 | 0.002132 | 0.331861 |
|  | 431 | naphthalene | 1.22223 | 0.049334 | 1.63E-07 |
|  | 18 | Methylmalonic acid | 1.18893 | 0.000126 | 3.940443 |
|  | 192 | glutaraldehyde 3 | 1.18109 | 0.037424 | 14.43508 |
|  | 438 | Glucose-1-phosphate | 1.14291 | 0.023284 | 2.797306 |
|  | 134 | 2'-deoxyguanosine 1 | 1.11628 | 0.012553 | 0.201356 |
|  | 657 | alpha-Tocopherol | 1.11312 | 0.005879 | 2.302931 |
|  | 661 | Zymosterol 2 | 1.10702 | 0.021748 | 2.20252 |
|  | 31 | Analyte 36 | 1.10363 | 0.031925 | 0.212533 |
|  | 380 | taurine | 1.09976 | 0.032933 | 0.45871 |
|  | 292 | Analyte 338 | 1.08944 | 0.028249 | 0.29113 |
|  | 248 | spermidine 1 | 1.05532 | 0.005149 | 0.815583 |
|  | 210 | Dehydroascorbic Acid 2 | 1.01511 | 0.017867 | 0.459833 |
|  | 625 | arachidonic acid | 1.00735 | 0.014212 | 0.362254 |
|  | 322 | 2,4-diaminobutyric acid 3 | 1.00648 | 0.032534 | 0.45332 |

**Table S-4.** Diﬀerential metabolites of SPD vs O group filtered by VIP>1 and P-value<0.05.

| No. | Var ID (Primary) | PEAK | VIP | P-VALUE | FOLD CHANGE |
| --- | --- | --- | --- | --- | --- |
|  | 261 | Analyte 300 | 2.04572 | 1.26E-09 | 6.68E-08 |
|  | 444 | Cysteinylglycine 3 | 1.99866 | 5.76E-08 | 0.49753 |
|  | 516 | pentadecanoic acid | 1.99697 | 1.25E-07 | 3499369 |
|  | 14 | Halostachine 2 | 1.99583 | 3.64E-06 | 0.594573 |
|  | 272 | 2-deoxy-D-glucose 1 | 1.96579 | 0.000407 | 0.738787 |
|  | 150 | glycine 2 | 1.96414 | 3.57E-05 | 0.522226 |
|  | 570 | Diglycerol 2 | 1.94082 | 1.49E-05 | 1991042 |
|  | 498 | 1,5-Anhydroglucitol | 1.93413 | 2.17E-05 | 79.83712 |
|  | 65 | naphthalene | 1.92993 | 3.9E-05 | 0.611823 |
|  | 520 | shikimic acid | 1.89589 | 0.000154 | 0.528382 |
|  | 18 | Methylmalonic acid | 1.85052 | 0.000371 | 3.216315 |
|  | 519 | xanthine | 1.83541 | 6.96E-05 | 0.422539 |
|  | 133 | phosphate | 1.8219 | 1.85E-05 | 0.111775 |
|  | 640 | 2-Monoolein | 1.81885 | 0.000361 | 1.737516 |
|  | 637 | terephthalic acid | 1.8016 | 7.7E-05 | 0.10116 |
|  | 97 | Methylmalonic acid | 1.79889 | 3.23E-06 | 0.621915 |
|  | 334 | 21-hydroxypregnenolone 4 | 1.79269 | 0.000111 | 0.238543 |
|  | 288 | oxoproline | 1.78093 | 0.000489 | 38843511 |
|  | 98 | oxalic acid | 1.78092 | 1.91E-07 | 0.602011 |
|  | 132 | phosphate | 1.77225 | 0.000647 | 2.993509 |
|  | 78 | phosphomycin | 1.76964 | 1.83E-05 | 0.685718 |
|  | 30 | glycine 1 | 1.75468 | 2.85E-06 | 0.527631 |
|  | 7 | glycolic acid | 1.74783 | 4.05E-05 | 0.638032 |
|  | 160 | D-Glyceric acid | 1.74153 | 5.14E-05 | 0.425226 |
|  | 552 | glutathione 1 | 1.74018 | 3.22E-05 | 0.596086 |
|  | 608 | Isoleucine | 1.73539 | 0.000573 | 7760672 |
|  | 148 | proline | 1.73033 | 0.000146 | 0.509782 |
|  | 221 | thymine | 1.72914 | 4.47E-05 | 0.651169 |
|  | 502 | Gluconic lactone 2 | 1.72184 | 0.001385 | 0.462993 |
|  | 59 | Farnesal 1 | 1.71896 | 0.00019 | 0.434064 |
|  | 507 | 5-Dihydrocortisol 1 | 1.71521 | 0.003059 | 0.724268 |
|  | 459 | 1,5-Anhydroglucitol | 1.71409 | 0.000749 | 6326856 |
|  | 176 | N-Oleoyldopamine 1 | 1.71344 | 0.000345 | 0.150423 |
|  | 350 | Glucose-1-phosphate | 1.71148 | 2.12E-05 | 0.498573 |
|  | 250 | phenylethylamine | 1.71145 | 7.04E-05 | 0.761582 |
|  | 191 | glutamine 1 | 1.70566 | 0.000993 | 19961183 |
|  | 483 | Methyl Palmitoleate | 1.69482 | 0.000371 | 0.67168 |
|  | 128 | urea | 1.69354 | 0.001457 | 1.04E+09 |
|  | 648 | Turanose 1 | 1.68956 | 0.001689 | 2.021203 |
|  | 262 | N-Oleoyldopamine 1 | 1.68618 | 0.001658 | 5.540296 |
|  | 335 | Dehydroabietic Acid | 1.68155 | 0.000924 | 0.438854 |
|  | 352 | phenylalanine 1 | 1.66434 | 0.000102 | 0.592164 |
|  | 90 | N-Ethylglycine 2 | 1.6637 | 0.004253 | 0.7124 |
|  | 659 | Farnesal 1 | 1.66229 | 0.001 | 1.16E-07 |
|  | 162 | 2-aminophenol 2 | 1.65326 | 0.000109 | 0.642193 |
|  | 539 | malonic acid 1 | 1.65 | 0.000488 | 0.323897 |
|  | 537 | myo-inositol | 1.64312 | 0.000271 | 0.612285 |
|  | 538 | Linoleic acid methyl ester | 1.64199 | 5.5E-05 | 0.684952 |
|  | 476 | mannose 1 | 1.64027 | 0.002396 | 0.579297 |
|  | 279 | noradrenaline | 1.62783 | 2.22E-05 | 0.794726 |
|  | 278 | asparagine 4 | 1.62655 | 0.000195 | 0.518308 |
|  | 142 | Isoleucine | 1.62436 | 0.00059 | 0.530278 |
|  | 463 | Prostaglandin E2 1 | 1.62189 | 3.8E-05 | 0.67799 |
|  | 9 | caprylic acid | 1.62066 | 0.00011 | 0.597269 |
|  | 105 | N-Oleoyldopamine 1 | 1.62064 | 0.000518 | 0.358513 |
|  | 564 | linoleic acid | 1.61666 | 0.001976 | 6086804 |
|  | 548 | methyl heptadecanoate | 1.61221 | 0.002546 | 0.332156 |
|  | 495 | tyrosine 1 | 1.60593 | 0.004421 | 0.565964 |
|  | 248 | spermidine 1 | 1.60308 | 0.010483 | 0.802574 |
|  | 11 | Analyte 13 | 1.60123 | 0.000369 | 0.371825 |
|  | 302 | Threonic acid | 1.59914 | 0.001064 | 0.564128 |
|  | 428 | glutamine 1 | 1.59715 | 0.004511 | 0.610813 |
|  | 37 | 3-Cyanoalanine | 1.5926 | 0.002752 | 0.542779 |
|  | 337 | thymidine 5'-monophosphate 1 | 1.58391 | 0.000399 | 0.597572 |
|  | 204 | MALONAMIDE 5 | 1.58316 | 0.000713 | 0.401296 |
|  | 358 | Glucose-1-phosphate | 1.57844 | 0.00015 | 0.524319 |
|  | 578 | oxalic acid | 1.5742 | 0.000331 | 0.501714 |
|  | 127 | benzoic acid | 1.57299 | 2.73E-05 | 0.658921 |
|  | 512 | isocitric acid 2 | 1.57265 | 0.000316 | 0.506515 |
|  | 115 | 2-Deoxyerythritol | 1.57111 | 0.002577 | 0.080624 |
|  | 551 | noradrenaline | 1.55611 | 0.005476 | 2.92928 |
|  | 653 | inosine 5'-monophosphate | 1.55012 | 0.003418 | 5619300 |
|  | 453 | oxalic acid | 1.53847 | 0.00051 | 0.467411 |
|  | 407 | valine | 1.53677 | 0.000639 | 0.657858 |
|  | 4 | p-benzoquinone | 1.53145 | 0.004401 | 0.475862 |
|  | 377 | Lyxose 1 | 1.52637 | 0.000151 | 0.569807 |
|  | 617 | N-epsilon-Acetyl-L-lysine 2 | 1.52614 | 0.011507 | 1.249866 |
|  | 186 | D-alanyl-D-alanine 1 | 1.52502 | 0.000848 | 0.700555 |
|  | 540 | malonic acid 1 | 1.51473 | 0.006054 | 0.428745 |
|  | 342 | ornithine | 1.50463 | 0.00201 | 0.439832 |
|  | 100 | valine | 1.50051 | 0.002327 | 0.570188 |
|  | 414 | putrescine 2 | 1.48962 | 0.000491 | 0.494793 |
|  | 16 | creatine degr | 1.4891 | 0.01318 | 0.629533 |
|  | 129 | glycerol | 1.48479 | 0.00365 | 0.387526 |
|  | 188 | Ethanolamine | 1.48122 | 3.74E-05 | 0.804944 |
|  | 315 | Corticosterone 2 | 1.47995 | 0.006534 | 0.421783 |
|  | 306 | L-cysteine | 1.47212 | 0.006313 | 0.478026 |
|  | 473 | glucose 2 | 1.46715 | 0.005938 | 0.583189 |
|  | 415 | creatine degr | 1.46408 | 0.000168 | 0.619617 |
|  | 68 | Ethanolamine | 1.46403 | 0.000146 | 0.670632 |
|  | 482 | mannose 2 | 1.46206 | 0.009899 | 0.604403 |
|  | 70 | Synephrine 2 | 1.45744 | 0.000641 | 0.621922 |
|  | 106 | cycloleucine 1 | 1.45639 | 0.000138 | 0.662947 |
|  | 477 | creatine degr | 1.4514 | 0.003973 | 0.488362 |
|  | 573 | ribose-5-phosphate 1 | 1.44573 | 0.011424 | 19475164 |
|  | 505 | Tricetin | 1.44168 | 0.003209 | 0.410326 |
|  | 31 | Analyte 36 | 1.43829 | 0.011343 | 0.098294 |
|  | 161 | acetanilide 1 | 1.43791 | 0.006654 | 0.495676 |
|  | 515 | glutamine 5 | 1.4371 | 0.008554 | 0.758749 |
|  | 378 | asparagine 1 | 1.43596 | 0.001613 | 0.578819 |
|  | 173 | 1-Methylhydantoin 2 | 1.43469 | 0.003759 | 0.700545 |
|  | 449 | conduritol b epoxide 2 | 1.43467 | 0.012439 | 6736286 |
|  | 641 | 2-Monopalmitin | 1.43357 | 0.011045 | 9143316 |
|  | 172 | uracil | 1.43318 | 0.001064 | 0.612714 |
|  | 12 | 5-Dihydrocortisol 2 | 1.43249 | 0.011236 | 0.783733 |
|  | 34 | Methylmalonic acid | 1.42063 | 0.0208 | 4.525798 |
|  | 25 | oxalic acid | 1.41691 | 0.003679 | 0.352031 |
|  | 443 | hypoxanthine 1 | 1.41659 | 0.000422 | 0.60204 |
|  | 84 | oxalic acid | 1.41415 | 0.000294 | 0.755887 |
|  | 452 | Gluconic lactone 2 | 1.40761 | 0.000822 | 0.531153 |
|  | 286 | methionine 1 | 1.40125 | 0.001236 | 0.577697 |
|  | 437 | 3-phosphoglycerate | 1.39868 | 0.005125 | 0.306706 |
|  | 316 | fructose 1 | 1.38678 | 0.001078 | 0.655581 |
|  | 21 | Citraconic acid degr1 | 1.38677 | 0.010127 | 0.625851 |
|  | 93 | ascorbate | 1.38327 | 0.000568 | 0.64652 |
|  | 503 | Pyrrole-2-Carboxylic Acid | 1.37548 | 0.003117 | 0.692084 |
|  | 216 | catechol | 1.36368 | 0.001385 | 0.820648 |
|  | 168 | picolinic acid | 1.36294 | 0.001666 | 0.586991 |
|  | 193 | Carbobenzyloxy-L-leucine degr1 | 1.36219 | 0.022998 | 0.117222 |
|  | 22 | alanine 1 | 1.35942 | 0.010892 | 0.710468 |
|  | 635 | Diglycerol 2 | 1.35853 | 0.015792 | 0.487053 |
|  | 247 | 4-aminobutyric acid 2 | 1.35546 | 0.016341 | 0.521005 |
|  | 536 | Farnesal 3 | 1.35201 | 0.000111 | 0.728578 |
|  | 33 | Analyte 38 | 1.35099 | 0.000836 | 0.639502 |
|  | 126 | oxamic acid | 1.34943 | 0.025943 | 0.111613 |
|  | 383 | creatine degr | 1.34814 | 0.006864 | 0.449636 |
|  | 260 | 9-Fluorenone 1 | 1.34303 | 0.020278 | 4.929278 |
|  | 312 | 3-phenylcatechol | 1.33523 | 0.004402 | 0.488002 |
|  | 156 | 2,3-Dihydroxypyridine | 1.33414 | 0.004583 | 0.471401 |
|  | 58 | xanthotoxin 1 | 1.33328 | 0.01006 | 0.351311 |
|  | 509 | creatine degr | 1.33153 | 0.003416 | 0.545869 |
|  | 547 | galactose 2 | 1.33117 | 0.026033 | 2.806082 |
|  | 517 | pentadecanoic acid | 1.32463 | 0.00687 | 0.290049 |
|  | 556 | 3,5-Dihydroxyphenylglycine 2 | 1.319 | 0.006766 | 0.583453 |
|  | 391 | xylitol | 1.3136 | 0.007631 | 0.586373 |
|  | 50 | Methylmalonic acid | 1.30561 | 0.011814 | 0.4168 |
|  | 399 | tetracosane | 1.30406 | 0.002685 | 0.574965 |
|  | 550 | 1,5-Anhydroglucitol | 1.30322 | 0.012542 | 0.223785 |
|  | 527 | Biphenyl | 1.29955 | 0.010951 | 0.566173 |
|  | 606 | Adipamide 1 | 1.29754 | 0.001514 | 0.733516 |
|  | 213 | Gluconic lactone 3 | 1.28551 | 0.040292 | 1.17E-07 |
|  | 194 | glucose 1 | 1.28441 | 0.018686 | 0.289663 |
|  | 26 | hydroxylamine | 1.2829 | 0.005292 | 0.537172 |
|  | 301 | Corticosterone 2 | 1.28157 | 0.031036 | 0.801248 |
|  | 292 | Analyte 338 | 1.27475 | 0.018778 | 0.299574 |
|  | 253 | beta-Glutamic acid 1 | 1.27222 | 0.001406 | 0.841647 |
|  | 481 | 2'-deoxyadenosine | 1.26937 | 0.003179 | 0.620867 |
|  | 295 | Purine riboside | 1.26925 | 0.001732 | 0.695367 |
|  | 400 | ribitol | 1.26135 | 0.027872 | 0.253608 |
|  | 77 | epsilon-Caprolactam | 1.2583 | 0.022276 | 0.710153 |
|  | 61 | Methylmalonic acid | 1.25761 | 0.027077 | 0.369798 |
|  | 76 | malonic acid 1 | 1.25647 | 0.018721 | 0.285157 |
|  | 489 | sorbitol | 1.25457 | 0.037217 | 9.805207 |
|  | 397 | N(epsilon)-Trimethyllysine | 1.25082 | 0.035318 | 0.515296 |
|  | 196 | N-methylaniline | 1.24542 | 0.011236 | 0.407601 |
|  | 406 | glutamic acid | 1.24379 | 0.033192 | 0.087746 |
|  | 57 | Cysteinylglycine 2 | 1.23865 | 0.012998 | 0.469321 |
|  | 23 | N-(3-aminopropyl)-morpholine 2 | 1.23505 | 0.000385 | 0.736796 |
|  | 166 | Methylmalonic acid | 1.2303 | 0.007277 | 0.779298 |
|  | 445 | ornithine 1 | 1.22116 | 0.023957 | 0.702927 |
|  | 32 | 2-hydroxybutanoic acid | 1.22005 | 0.018564 | 0.408709 |
|  | 485 | lysine | 1.21775 | 0.035218 | 0.619052 |
|  | 66 | 4-HYDROXYPYRIDINE | 1.2161 | 0.003233 | 0.784791 |
|  | 121 | cis-Phytol | 1.21439 | 0.01772 | 0.571917 |
|  | 140 | citrulline 2 | 1.2135 | 0.020782 | 0.728976 |
|  | 190 | Pipecolinic acid | 1.20657 | 0.016922 | 0.401283 |
|  | 271 | asparagine 4 | 1.20148 | 0.026798 | 0.363534 |
|  | 514 | Glucose-1-phosphate | 1.18268 | 0.01669 | 0.675252 |
|  | 479 | tyrosine 2 | 1.18082 | 0.041454 | 0.374411 |
|  | 403 | beta-Glycerophosphoric acid | 1.17359 | 0.007139 | 0.614874 |
|  | 154 | N-methyltryptophan | 1.17108 | 0.02041 | 0.755094 |
|  | 508 | lactose 1 | 1.15691 | 0.019964 | 0.428694 |
|  | 584 | 2-Monoolein | 1.15532 | 0.037608 | 0.397534 |
|  | 225 | Aminomalonic acid | 1.15155 | 0.007023 | 0.668035 |
|  | 259 | L-Malic acid | 1.14224 | 0.038134 | 0.629985 |
|  | 72 | Methyl Phosphate | 1.14076 | 0.016884 | 0.57835 |
|  | 462 | noradrenaline | 1.13758 | 0.036991 | 0.528437 |
|  | 235 | 5-Methoxytryptamine 2 | 1.13303 | 0.001801 | 0.846117 |
|  | 457 | oxoproline | 1.12959 | 0.018576 | 0.547587 |
|  | 466 | asparagine 3 | 1.1283 | 0.037787 | 0.305686 |
|  | 177 | fumaric acid | 1.12488 | 0.040061 | 0.599348 |
|  | 54 | 3-hydroxybutyric acid | 1.11275 | 0.048521 | 0.510779 |
|  | 401 | Fluorene | 1.11103 | 0.029057 | 0.506793 |
|  | 405 | Analyte 466 | 1.09702 | 0.045425 | 0.320611 |
|  | 620 | Diglycerol 2 | 1.0932 | 0.035937 | 0.585906 |
|  | 430 | O-Phosphorylethanolamine | 1.09297 | 0.007356 | 0.718602 |
|  | 395 | urocanic acid 2 | 1.08399 | 0.017647 | 0.678194 |
|  | 273 | nicotinic acid | 1.0824 | 0.049962 | 0.538326 |
|  | 339 | Pipecolinic acid | 1.07277 | 0.012602 | 0.646865 |
|  | 2 | Mono(2-ethylhexyl)phthalate | 1.06655 | 0.001263 | 0.807615 |
|  | 130 | glycerol | 1.06009 | 0.005055 | 0.691826 |
|  | 201 | 4-Hydroxy-3-methoxybenzyl alcohol | 1.0464 | 0.039061 | 0.555658 |
|  | 252 | glycerol | 1.03767 | 0.005721 | 0.739525 |
|  | 40 | 5-Methoxytryptamine 1 | 1.0333 | 0.000441 | 0.83574 |
|  | 223 | 2-aminoethanethiol | 1.02925 | 0.002177 | 0.853202 |
|  | 472 | adenine | 1.02893 | 0.005781 | 0.781837 |
|  | 274 | nicotinamide | 1.02207 | 0.00354 | 0.759428 |
|  | 185 | Pelargonic acid | 1.01235 | 0.010254 | 0.779665 |
|  | 420 | D-(glycerol 1-phosphate) | 1.00777 | 0.029066 | 0.591069 |
|  | 152 | succinic acid | 1.00115 | 0.047647 | 0.758528 |

**Table S-5.** Pathway enrichment analysis for molecules from SP group.

| Pathway KEGG ID | Pathways name | Total*^a^* | Hit*^b^* | P-value*^c^* | matching Molecules in your pathway (IDs *^d^*) |
| --- | --- | --- | --- | --- | --- |
| ko04917 | Prolactin signaling pathway | 90 | 25 | 0 | K11220 K04692 |
| ko04610 | Coagulation and complement cascades | 87 | 12 | 1.08E-12 | K03903 K03990 K03989 K03912 K03904 K03905 K03917 K01313 K03898 K03910 |
| ko05146 | Amoebiasis | 108 | 7 | 1.19E-05 | K05717 K06237 K04000 |
| ko05150 | Staphylococcus aureus infection | 83 | 6 | 2.75E-05 | K03990 K03989 K04004 K03905 K01333 |
| ko04630 | Jak-STAT signaling pathway | 144 | 7 | 7.58E-05 | K11220 K04692 |
| ko00480 | Glutathione metabolism | 111 | 5 | 0.001175 | K00432 K00799 C00315 C05422 |
| ko05212 | Pancreatic cancer | 65 | 4 | 0.001178 | K11220 K04692 |
| ko04512 | ECM-receptor interaction | 263 | 7 | 0.002751 | K06237 K05717 K06238 |
| ko00330 | Arginine and proline metabolism | 201 | 6 | 0.003145 | C00300 K00294 C00086 C00148 C00315 |
| ko05143 | African trypanosomiasis | 49 | 3 | 0.005089 | K13822 K14477 |
| ko00010 | Glycolysis / Gluconeogenesis | 157 | 5 | 0.005283 | K12406 K01689 K01834 |
| ko04210 | Apoptosis | 138 | 4 | 0.01695 | K06114 K01379 K02310 |
| ko00052 | Galactose metabolism | 146 | 4 | 0.020394 | C00243 C00089 K00011 K00963 |
| ko04062 | Chemokine signaling pathway | 152 | 4 | 0.02324 | K11220 K04692 |
| ko05200 | Pathways in cancer | 428 | 7 | 0.033208 | K11220 K04692 |
| ko00230 | Purine metabolism | 627 | 9 | 0.034836 | K01490 K12406 C00086 C00330 K00364 K01509 |
| ko05145 | Toxoplasmosis | 109 | 3 | 0.042734 | K11220 K04692 |

*^a^* The total number of molecules in the pathway.

*^b^* The number of molecules derived from omics analysis.

*^c^* Probability of identified differential protein list associates to the generated pathway.

*^d^* ID from KEGG database.

**Table S-6.** Pathway enrichment analysis for molecules from SPD group.

| Pathway KEGG ID | Pathways name | Total*^a^* | Hit*^b^* | P-value*^c^* | matching Molecules in your pathway (IDs *^d^*) |
| --- | --- | --- | --- | --- | --- |
| ko00480 | Glutathione metabolism | 111 | 22 | 0 | C01419 C01879 C00025 K00799 C00077 C00097 C00134 |
| ko04610 | Coagulation and complement cascades | 87 | 13 | 8.49E-14 | K03903 K03984 K03990 K03989 K03912 K03904 K01335 K03905 K01313 K03898 K03910 |
| ko00052 | Galactose metabolism | 146 | 14 | 4.68E-12 | C00794 C00243 C00124 C00031 C00116 K00011 |
| ko00250 | Alanine, aspartate and glutamate metabolism | 110 | 8 | 1.77E-06 | C00064 C00025 |
| ko00471 | D-Glutamine and D-glutamate metabolism | 20 | 4 | 1.35E-05 | C00064 C00025 |
| ko05150 | Staphylococcus aureus infection | 83 | 6 | 3.78E-05 | K03990 K03989 K04004 K03905 K01333 |
| ko00330 | Arginine and proline metabolism | 201 | 8 | 0.00014 | C00300 C00086 C00025 C00077 K00274 C00134 |
| ko00910 | Nitrogen metabolism | 156 | 7 | 0.000178 | C00064 K01672 C00025 |
| ko00260 | Glycine, serine and threonine metabolism | 158 | 7 | 0.000192 | C00258 C00300 C00097 C00197 K00274 K01834 |
| ko00970 | Aminoacyl-tRNA biosynthesis | 122 | 5 | 0.002286 | C00407 C00064 C00025 C00082 C00097 |
| ko00561 | Glycerolipid metabolism | 132 | 5 | 0.003215 | C00258 K00011 C00116 C00197 |
| ko05143 | African trypanosomiasis | 49 | 3 | 0.005949 | K13822 K14477 |
| ko00010 | Glycolysis / Gluconeogenesis | 157 | 5 | 0.00668 | K01689 C00031 C00197 K01834 |
| ko00680 | Methane metabolism | 285 | 6 | 0.020792 | C00258 C00082 K01689 C00197 K01834 |
| ko05205 | Proteoglycans in cancer | 259 | 5 | 0.046068 | K05717 K06251 K06484 |
| ko00230 | Purine metabolism | 627 | 9 | 0.04711 | C00130 C00086 C00064 C00385 K00364 K01509 |

*^a^* The total number of molecules in the pathway.

*^b^* The number of molecules derived from omics analysis.

*^c^* Probability of identified differential protein list associates to the generated pathway.

*^d^* ID from KEGG database.
